# A drug repurposing strategy for overcoming human multiple myeloma resistance to standard-of-care treatment

**DOI:** 10.1038/s41419-022-04651-w

**Published:** 2022-03-04

**Authors:** Katarina Chroma, Zdenek Skrott, Jan Gursky, Jaroslav Bacovsky, Pavel Moudry, Tereza Buchtova, Martin Mistrik, Jiri Bartek

**Affiliations:** 1 Laboratory of Genome Integrity, Institute of Molecular and Translational Medicine, Faculty of Medicine and Dentistry, Palacky University, Olomouc, Czech Republic; 2 Department of Hemato-oncology, University Hospital Olomouc and Medical Faculty of Palacky University Olomouc, Olomouc, Czech Republic; 3 Danish Cancer Society Research Center, Copenhagen, Denmark; 4 Division of Genome Biology, Department of Medical Biochemistry and Biophysics, Science for Life Laboratory, Karolinska Institute, Stockholm, Sweden

**Keywords:** Myeloma, Cancer therapeutic resistance

## Abstract

Despite several approved therapeutic modalities, multiple myeloma (MM) remains an incurable blood malignancy and only a small fraction of patients achieves prolonged disease control. The common anti-MM treatment targets proteasome with specific inhibitors (PI). The resulting interference with protein degradation is particularly toxic to MM cells as they typically accumulate large amounts of toxic proteins. However, MM cells often acquire resistance to PIs through aberrant expression or mutations of proteasome subunits such as PSMB5, resulting in disease recurrence and further treatment failure. Here we propose CuET—a proteasome-like inhibitor agent that is spontaneously formed in-vivo and in-vitro from the approved alcohol-abuse drug disulfiram (DSF), as a readily available treatment effective against diverse resistant forms of MM. We show that CuET efficiently kills also resistant MM cells adapted to proliferate under exposure to common anti-myeloma drugs such as bortezomib and carfilzomib used as the first-line therapy, as well as to other experimental drugs targeting protein degradation upstream of the proteasome. Furthermore, CuET can overcome also the adaptation mechanism based on reduced proteasome load, another clinically relevant form of treatment resistance. Data obtained from experimental treatment-resistant cellular models of human MM are further corroborated using rather unique advanced cytotoxicity experiments on myeloma and normal blood cells obtained from fresh patient biopsies including newly diagnosed as well as relapsed and treatment-resistant MM. Overall our findings suggest that disulfiram repurposing particularly if combined with copper supplementation may offer a promising and readily available treatment option for patients suffering from relapsed and/or therapy-resistant multiple myeloma.

## Introduction

Multiple myeloma (MM), the second most frequent hematologic malignancy represents a still incurable plasma cell disease [1, 2]. MM cells originating from antibody-producing B cells usually maintain immunoglobulin production, thereby causing endogenous proteotoxic stress and sensitivity to drugs targeting protein degradation. For many MM patients, the first-line therapy includes the proteasome inhibitor (PI) bortezomib, which significantly improves patients’ outcomes. However, disease relapse usually follows in bortezomib-treated patients. To overcome the acquired bortezomib resistance, the second and third generation of PIs were developed, including carfilzomib and ixazomib, or marizomib and oprozomib currently undergoing clinical evaluation. While these drugs differ from each other by pharmacodynamic properties or route of administration, all target the 20 S proteasome, raising the possibility of cross-resistance among them if the mode of resistance reflects mutations of active proteasome subunits (e.g. PSMB5) or altered expression of proteasome subunit [3, 4]. To overcome this limitation, alternative approaches to target proteasome or more generally the ubiquitin-proteasome degradation system (UPS) are under pre-clinical testing for MM treatment. These approaches include targeting 19 S proteasome components such as deubiquitinases POH1 [5] and USP14/UCHL5 [6] or ubiquitin-binding receptor RPN13 [7]. These inhibitors were effective against MM in vitro and in vivo, induced accumulation of ubiquitinated proteins, and activation of the cellular unfolded protein response (UPR) – a stress pathway leading to cell death [8–10]. The UPS machinery can be therapeutically targeted also at other steps besides the proteasome, by inhibition of the ubiquitin-activating enzyme (UEA1) by TAK-243 [11] or inhibition of p97/VCP segregase upstream of the proteasome - by CB5083 [12]. Both these compounds showed robust anti-MM activities in vitro and in vivo, accompanied by accumulation of non-degraded proteins and activation of the UPR [13–15]. While these molecular targets could possibly overcome the bortezomib/carfilzomib resistance caused by mutations in the proteasome subunits, such prediction is uncertain concerning other modes of resistance. Moreover, the point mutation within proteasome subunits abolishing bortezomib activity was so far found only in one study [4] but not by others [16, 17] suggesting the rarity of nonrecurring mutations or polyploidy of proteasome subunits [18]. Accumulating evidence indicates that therapy resistance can reflect complex metabolic changes and clonal evolution of MM cells. For example treatment resistance in some MM patients can reflect expansion of Xbp1s^low^ B tumor cells and pre-plasmablasts which are intrinsically resistant to bortezomib due to lower immunoglobulin production and decreased proteasome load [19], thus supporting the load-versus-capacity theory [20]. Others confirmed a correlation of the low-level XBP1s and immunoglobulin production with poor responses to bortezomib therapy [21] or decreased sensitivity in vitro [22]. In terms of the MM secretory status, patients with measurable disease responded to bortezomib better than oligo- or non-secretory MM patients [23] consistent with Ig production/secretion sensitizing MM to bortezomib [24]. Some studies reported MM cell dedifferentiation and clonal propagation of pre-plasma cells during therapy [25, 26]. In such cases of bortezomib resistance, it is unclear whether alternative approaches of targeting the proteasome or UPS, in general, might be effective because such treatments still rely on the induction of ER stress and UPR-mediated cell death, scenarios potentially difficult to trigger in dedifferentiated, less secretory pre-plasma MM cells.

Here, we assessed Disulfiram (Antabuse), a well-tolerated alcohol-abuse drug and a promising candidate for repurposing in oncology [27–30]. In the context of MM, DSF might be particularly interesting because its anticancer activity is related to the CuET metabolite which is spontaneously formed in-vivo and in-vitro in the presence of copper ions [31]. CuET shows proteasome-like inhibitory effects *via* aggregation of NPL4 (an essential adaptor of p97) and induction of UPR in a broad range of cell lines [31]. We previously reported anti-MM efficacy of CuET for both in-vitro and in-vivo models [31]. In this study, we examine whether CuET may act similarly to the p97 inhibitor CB5083 (not approved clinically) and overcome resistance to proteasome inhibitors mediated by mutations of active proteasome subunits (e.g. PSMB5) or altered expression of proteasome subunits. Moreover, as CuET induces aggregation of NPL4 and some other proteins directly, we propose that it might trigger the UPR independently of the extent of endogenous proteotoxic stress and/or disbalance of other, non-UPS cancer-relevant pathways, such as through triggering replication stress which was also reported among CuET-induced effects [32].

## Results

### CuET immobilizes NPL4, activates UPR and HSP in MM cell lines

**Fig. 1:**
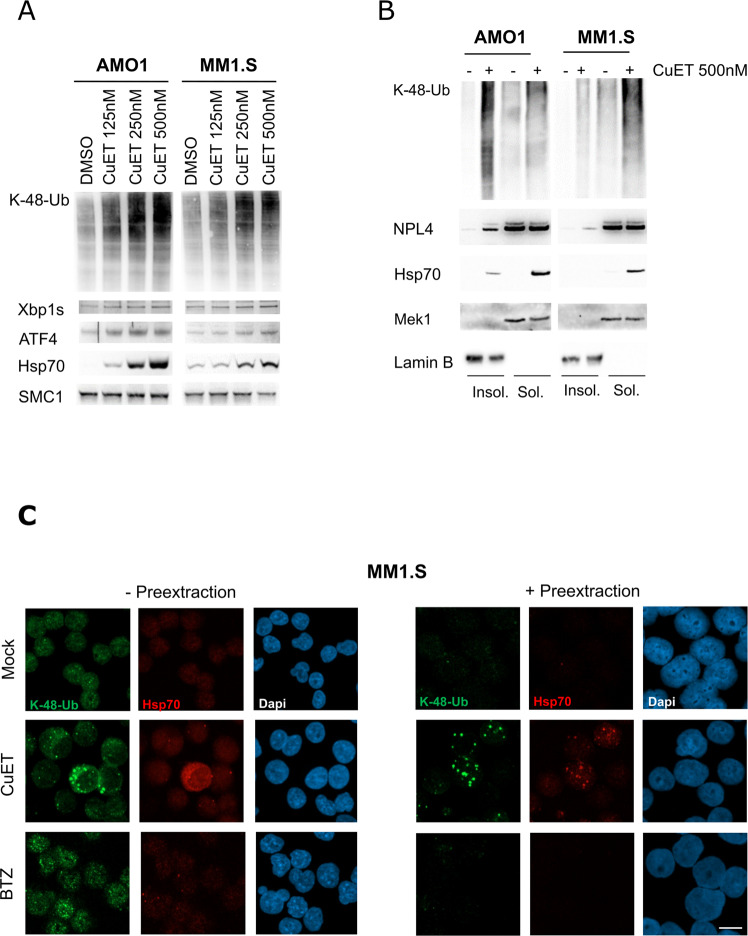
Exposure to submicromolar CuET induces proteotoxic stress, protein aggregation, and activation of UPR and heat-shock pathways in MM cell lines. **A** CuET induces poly-ubiquitinated proteins (K-48-Ub), increases XBP1s and ATF4 proteins (markers of unfolded protein response, UPR), and accumulates heat shock protein HSP70 in AMO1 and MM1.S myeloma cell lines. **B** Immobilization of NPL4, HSP70, and K48-Ub- proteins in Triton X-100 pre-extracted pellet fraction of CuET–treated (500 nM, 5 h) AMO1 and MM1.S cells. **C** Immunofluorescence analysis of CuET (500 nM) and BTZ (10 nM) induced K48- poly-Ub proteins together with HSP70 in MM cell lines without/with pre-extraction step (5 h treatment). Scale 10 μM.

Previously, we reported higher CuET sensitivity of myeloma cells than cell lines from other types of cancer [31]. Here, we first verified the enhanced CuET responsiveness in human AMO1 and MM1.S myeloma cell lines compared with breast cancer-derived MDA-MB-231 and osteosarcoma U2OS cells (Supplementary Fig. S1A, S1B) which are good CuET responders [31]. To elucidate the mechanism behind the MM hypersensitivity we first assessed the endogenous levels of VCP/p97 and its adaptor NPL4, the known target of CuET [31]. Among a series of cancer cell lines (Supplementary Fig. S1C), the abundance of NPL4 was variable, yet without any obvious correlation explaining the sensitivity. Next, we compared the extent of endogenous proteotoxic stress using K48-ubiquitin (K48-Ub) smears as readouts. Both AMO1 and MM1.S cells showed elevated K48-Ub smears compared to MDA-MB-231 and U2OS cells suggesting a relationship to CuET-sensitivity (Supplementary Fig. S1C) as the p97/NPL4 pathway is directly involved in the processing of K48-ubiquitylated proteins [33]. As MM cells grow in suspension, we assessed whether CuET induces the same phenotypes as published for other cell lines including proteasome-inhibition-like response, UPR and heat-shock response (HSR) [31]. CuET-exposed MM cells indeed accumulate polyubiquitinated (poly-Ub) proteins in a dose-dependent manner (Fig. 1A), a feature typically shared with PIs such as bortezomib or MG132. Also, UPR components were elevated, including the spliced form of XBP1s and ATF4 and overabundant chaperone HSP70, the key HSR component, in both CuET-treated MM cell lines (Fig. 1B). Next, we asked whether CuET immobilizes NPL4 in MM cells, as reported for U2OS and MDA-MB-231 cells [31]. Indeed, the CuET-induced immobilization of endogenous NPL4 was confirmed by immunoblotting analysis of detergent-insoluble cellular fractions in both MM cell lines (Fig. 1B). Consistently with published data, the insoluble fractions were also enriched for polyubiquitinated proteins and HSP70 (Fig. 1B).

Accumulation of poly-Ub proteins, UPR, and HSR activation are features of CuET treatment shared with PIs [34] clinically used for MM treatment [35]. However, the observed enrichment of insoluble poly-Ub proteins and HSP70 seems to be a unique effect induced only by CuET. This effect is well apparent by immunofluorescence analysis where the increased proteotoxic stress (elevated K48-Ub) and HSP70 are detectable for CuET and BTZ in non-pre-extracted cells but upon Triton-X-100 pre-extraction, only CuET-treated cells show such signals (Fig. 1C). Collectively, these data indicate that in AMO1 and MM1.S MM models, CuET induces phenotypes shared with adherent cancer cell lines, and despite these phenotypes share some features with PI effects, there are also responses unique to CuET.

### CuET toxicity against MM is unaffected by *PSMB5* mutation responsible for resistance to PI

**Fig. 2:**
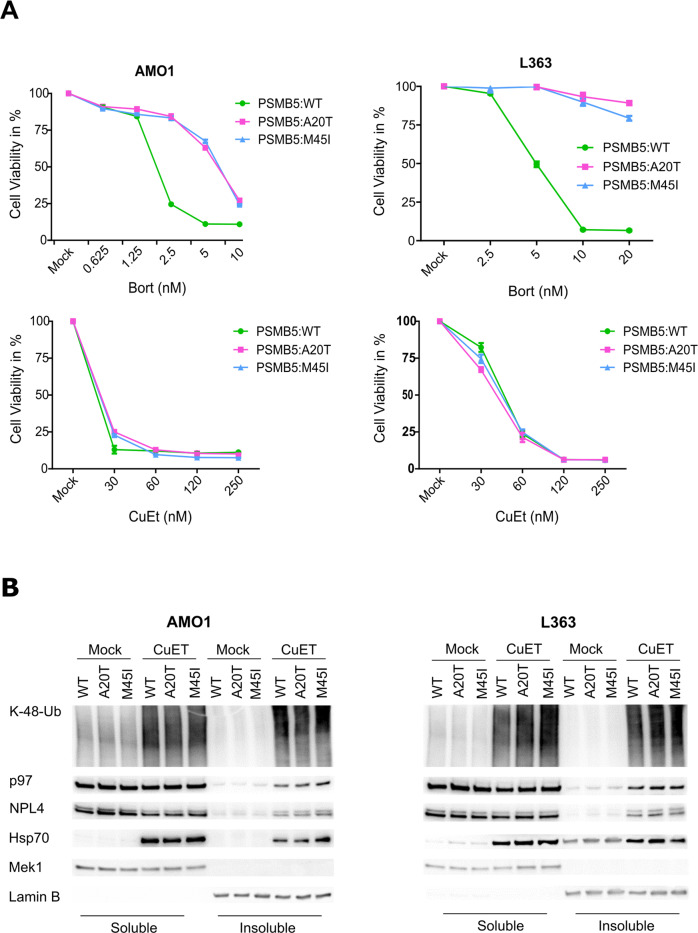
*PSMB5* mutations do not protect MM cells from CuET toxicity. **A** XTT-based viability test after 72 h treatment of indicated concentrations of BTZ or CuET in AMO1 (left) and L363 (right) sublines with mutated *PSMB5* proteasome subunit (A20T, M45I) compared to the WT counterparts. Data are mean ± s.d. of three independent experiments. **B** CuET induces immobilization of NPL4, P97, poly-Ub proteins, and HSP70 in the insoluble fraction of WT, as well as PSMB5, mutated sublines of AMO1 and L363.

DSF reacts with copper ions in-vitro and in-vivo, ultimately forming the active metabolite CuET with PI-like activity [36]. Despite some reports suggesting proteasome as the direct target of DSF/Copper treatment, we demonstrated that neither 20 S nor 26 S proteasome is affected by DSF or CuET [31]. Instead, CuET acts upstream of the proteasome by interference with the p97/NPL4 segregase involved in pre-processing of proteins destined for degradation [31]. Consistent with such mode of action, any acquired resistance that involves proteasome modification should not affect the sensitivity of MM cells towards CuET. Recently, Barrio and colleagues [4] identified three somatic point mutations in proteasome subunit *PSMB5* as the underlying cause for resistance in MM cells chronically exposed to BTZ. We obtained two such cell lines including Bortezomib (Carfilzomib, Ixazomib)-resistant variants of AMO1 and L363 [4]. The wild-type (wt) AMO1 and L363 cell lines together with their sublines bearing two somatic *PSMB5* mutations (A20T, M45I) were tested for sensitivity to BTZ and CuET. As expected, overexpression of mutant *PSMB5* in either cell line induced BTZ resistance (Fig. 2A). In contrast, CuET decreased the cell viability regardless of the mutations (Fig. 2A), increased K48-Ub in the *PSMB5*-mutant cell lines similarly to the wild-type and also induced the same amount of immobilized poly-Ub proteins. The insoluble fraction was also enriched similarly for NPL4, P97, and HSP70 in the wt and mutant models (Fig. 2B). These results show that the CuET-induced 20 S proteasome-independent blockade of the proteolysis machinery remains still effective in cells with the clinically relevant proteasome PSMB5 subunit mutations.

### The cytotoxic effect of CuET is unaffected by reduced protein synthesis

**Fig. 3:**
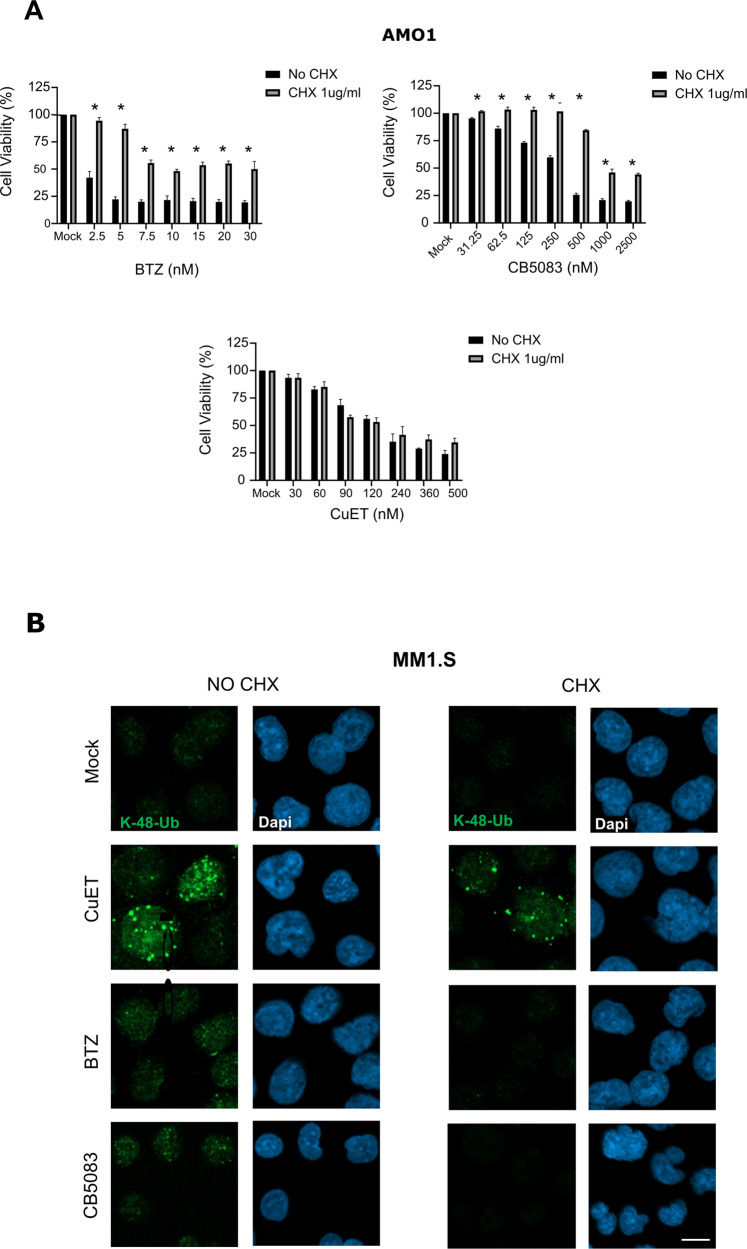
Protein synthesis rate does not affect the cytotoxic impact of CuET. **A** Co-treatment with cycloheximide (CHX) does not rescue AMO1 cell death in CuET- treated samples in contrast to BTZ and p97 inhibitor (CB5083). AMO1 cells were cultured for 24 h in the presence of the indicated doses of CuET, BTZ, and CB5083 in the presence or absence of 1 μg/ml CHX. Data are mean ± s.d. of three independent experiments. Statistical significance was determined using T-test, *P < 0,03. **B** CHX does not reduce CuET- induced accumulation of poly-Ub proteins when compared to BTZ, CB5083, and Mock (DMSO) treated samples. MM1.S were treated by 500 nM CuET, 2.5 nM BTZ or 10 μM CB5083 and co-treated with CHX 10 μg/ml. After 3 h the cells were either pre-extracted and fixed or directly fixed and stained for K48-Ub. Scale 10 μM.

**Fig. 4:**
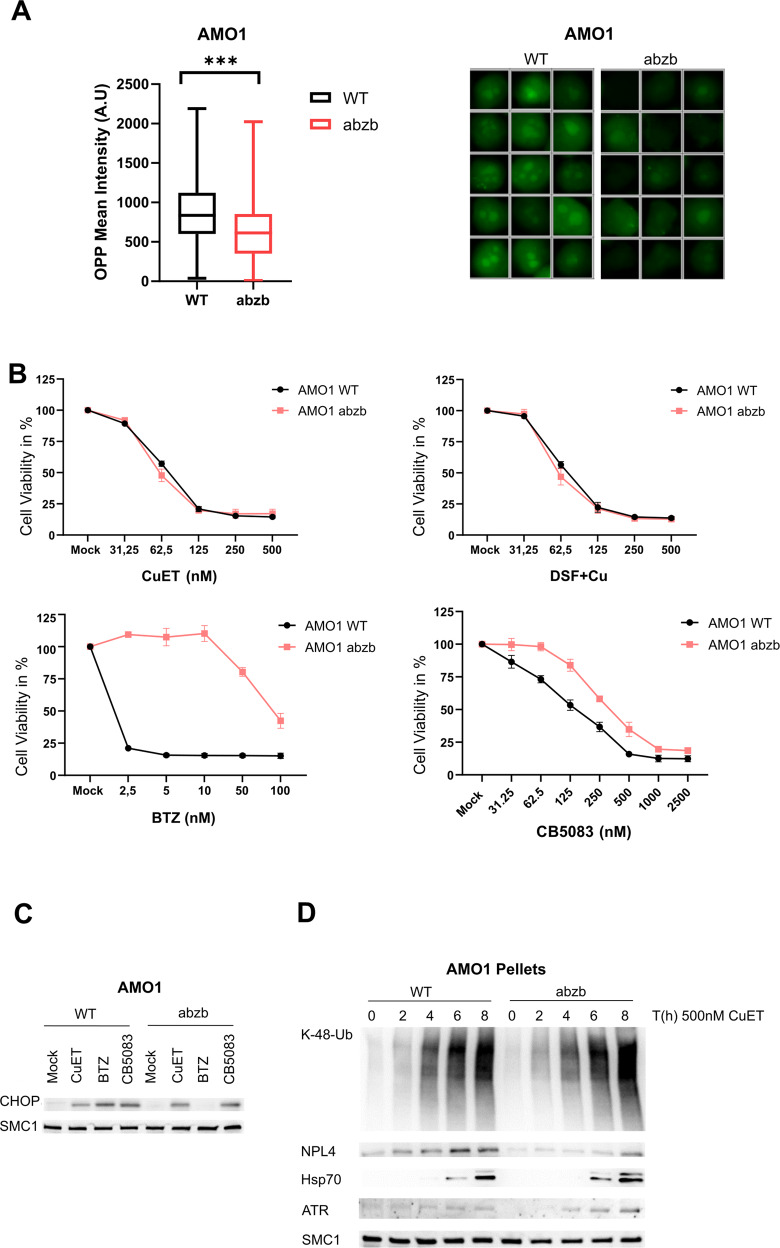
MM cells adapted to bortezomib via decreased proteosynthesis remain sensitive to CuET treatment. **A** Bortezomib-adapted (BTZ res) AMO1 abzb cell line has reduced protein biosynthesis compared to parental AMO1, measured by OPP-Click assay. Representative microscopic images from the quantification of the OPP-Click signal are included. Results are mean ± s.d. of three independent experiments. Statistical significance was determined using T-test, ***P ≤ 0,0001. **B** BTZ-adapted AMO1 abzb and non-adapted AMO1 cells are equally sensitive to treatment with CuET and DSF + Cu. Cells were cultured for 48 h with indicated doses of CuET, DSF, BTZ, and CB5083. **C** CuET and CB5083 activate the UPR pathway in AMO1 abzb similarly to the parental AMO1, measured by induction of pro-apoptotic protein CHOP. Cells were treated for 14 h with 250 nM CuET, 5 nM BTZ, and 250 nM CB5083 and subsequently lysed and probed for the level of CHOP protein. **D** CuET triggers the formation of protein aggregates in AMO1 abzb similar to the parental AMO1. Western blot detection of CuET- response associated K-48-Ub proteins, NPL4, HSP70 in Triton X-100 resistant protein fraction. Cells were treated with 500 nM CuET and lysed in indicated timepoints.

One of the suggested resistance mechanisms of MM cells towards PIs reflects attenuated proteosynthetic activity. In such settings, the clinically relevant PIs become inefficient because the proteasome workload/capacity and extent of protein synthesis are closely interconnected [20]. Notably, such a universal resistance mechanism of MM cells might impact also responses to inhibitors targeting the protein degradation upstream of proteasome, including p97 inhibitors. This idea has however never been tested. The proteosynthesis-based resistance mechanism can be experimentally tested in MM cell lines by co-treatment with cycloheximide (CHX), an inhibitor of protein synthesis at translational level [37]. Indeed, co-treatment with CHX and BTZ indeed rescued the bulk of AMO1 and MM1.S cells from BTZ-induced reduction of cell viability (Fig. 3A and Supplementary Fig. S2). The same rescue effect was detected also for CB5083 (a selective inhibitor of p97) (Fig. 3A and Supplementary Fig. S2). Surprisingly, in contrast to this, the protective effect of CHX was not observed in MM cells treated by CuET, where the drop of cell survival almost recapitulated the outcome of treatment by CuET alone (Fig. 3A and Supplementary Fig. S2). To elucidate this effect, we examined the accumulation of K48-linked poly-Ub proteins in single-compound treated and co-treated cells. Interestingly, only CuET accumulated K48-linked poly-Ub proteins in combination with CHX (Fig. 3B). Next, we employed yet another cellular model: the BTZ-adapted myeloma cell line AMO1-abzb, established by chronic exposure of AMO1 cells to bortezomib, displaying altered proteasome system and reduced proteo-synthesis [38]. First, we compared the AMO1-wt and AMO1-abzb cells’ proteo-synthesis levels using the OPP click assay. As expected, AMO1-abzb showed reduced overall proteo-synthesis (Fig. 4A) and fewer K-48 poly Ub-proteins (Supplementary Fig. S3) suggesting an overall lower proteotoxic stress. Next, we examined the sensitivity of the AMO1-wt *versus* AMO1-abzb cells towards BTZ, CB5083, and CuET. As expected, AMO1-abzb cells were resistant to BTZ and cross-resistant to CB5083, at least at lower concentrations (Fig. 4B). Strikingly, both cell lines showed similar concentration-dependent decrease of cell viability when treated by CuET (Fig. 4B). In contrast to BTZ, CuET and also CB5083 treatments of both parental and BTZ-resistant AMO1 led to UPR activation detected by enhanced UPR-associated proapoptotic protein Chop (Fig. 4C) accompanied by apoptosis (Supplementary Fig. S4). Furthermore, in both cell lines, CuET induced additional accumulation of K-48 poly Ub-proteins and enrichment of NPL4 and HSP70 in the insoluble fractions (Fig. 4D). Notably, the combined DSF + Cu treatment closely mimicked the cell survival effects obtained upon treatment with CuET (Fig. 4B), further confirming previous observations that these two treatment strategies are interchangeable [36].

### CuET kills CD319^+^ MM patients’ cells independently of disease stage and therapy

**Fig. 5:**
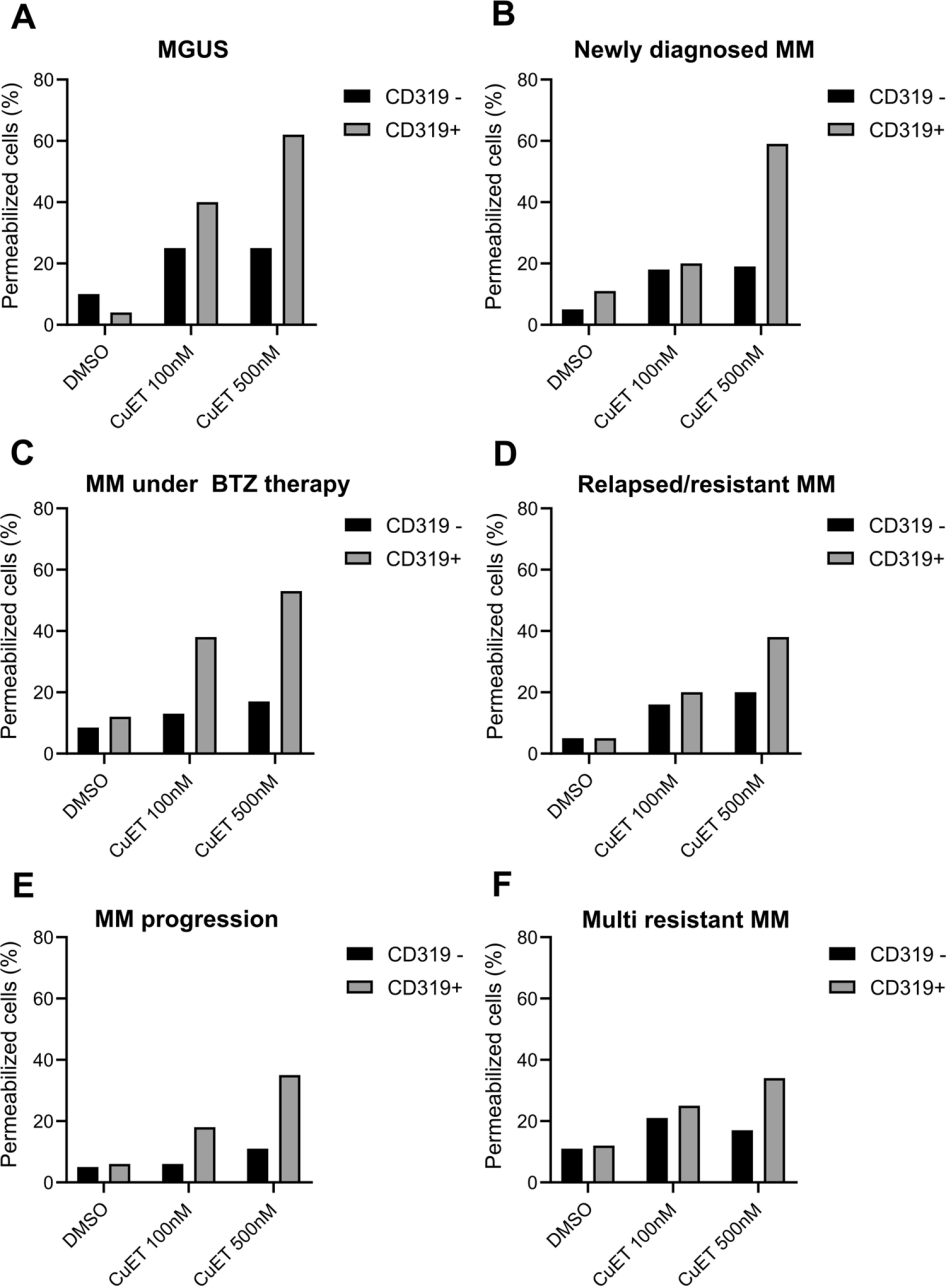
CuET efficiently kills CD319^+^/CD138^+^ MM cancer cells isolated from patients independently of disease stage and ongoing/passed therapy. **A** Cytotoxic effect of CuET in a patient diagnosed for Monoclonal gammopathy of undetermined significance (MGUS; asymptomatic Multiple Myeloma). **B** Cytotoxic effect of CuET in a patient newly diagnosed with Multiple Myeloma without any passed therapy. **C** Cytotoxic effect of CuET in a patient diagnosed with Multiple Myeloma under Bortezomib therapy. **D** Cytotoxic effect of CuET in relapsed Multiple Myeloma patients after two years in remission. **E** Cytotoxic effect of CuET in a patient been in progression phase of the disease after a remission. **F** Cytotoxic effect of CuET in Multiple Myeloma patients being resistant to all available anti-MM therapies (proteasome inhibitors and immunomodulatory drugs). Each graph represents a single patient, samples treated for 24 h.

To validate the specific cytotoxic effect of CuET in MM seen in cell culture models also in clinical settings, we examined bone marrow samples from 14 patients (male and female) diagnosed with MM or Monoclonal gammopathy of undetermined significance (MGUS). The ex-vivo testing setup involved depletion of red blood cells from fresh bone marrow biopsies followed by CuET treatment (100 nM and 500 nM for 24 h). In each sample, the MM cells were identified by flow cytometry using anti-CD-319-PE and anti-CD-138-APC antibodies [39–42]. The separated noncancerous (CD319^-^) and cancerous (CD319^+^) cell fractions were examined for cell viability using plasma membrane permeabilization (DAPI positive). These results showed cytotoxic effect of CuET preferentially against the CD319^+^ MM cells independently of disease stage (MGUS or MM) and ongoing therapy (Fig. 5A–F). Thus, CuET induced cell death of CD319^+^ cells derived from patients diagnosed for MGUS (Fig. 5A), a patient diagnosed for MM having no previous therapy (Fig. 5B) as well as a MM patient under BTZ therapy (Fig. 5C). Importantly, we detected the cytotoxic effect of CuET also in the sample from a patient who relapsed after a 2-year disease-free period (Fig. 5D) and from two MM patients exhibiting resistance to all combinations of available clinically used anti-MM drugs (Fig. 5E, F). The latter included prior combinatory therapy by PIs, immunomodulatory and alkylating antineoplastic drugs usually prescribed to resistant, relapsed, and refractory myeloma patients. Overall, this data not only identify CuET as a plausible new therapeutic strategy for patients diagnosed with MM independently of disease stage and therapy history but also highlight an innovative and promising treatment approach for patients currently regarded as incurable, resistant to the full spectrum of currently available anti-MM drugs.

## Discussion

Proteasome inhibitors such as bortezomib constitute the first-line anti-MM therapy, however, acquired resistance occurs commonly [43]. Here, we provide evidence that the alcohol-abuse drug disulfiram could be a promising candidate for repurposing in treatment-resistant MM. The anticancer effect of DSF has been tested in numerous clinical trials [44]. From the mechanistic point of view, the hypothesis about DSF’s anticancer effect being linked to inhibition of aldehyde dehydrogenase [45] was recently refuted [36]. Instead, the DSF’s metabolite CuET represents the ultimate anticancer agent, explaining potentiation of DSF by copper [31]. Treatment of cells with CuET evokes phenotypes resembling inhibitors of protein degradation such as bortezomib or p97 [12, 31] via inhibition and aggregation of NPL4, an essential co-factor of p97 segregase [31, 46] involved in protein degradation upstream of the proteasome. Similar to other UPS blockers, CuET shows robust toxicity towards MM cells including preclinical models [31, 47]. Based on the rationale that CuET impairs UPS outside the proteasome we addressed here its toxic effect using the recently described clinically-relevant BTZ-resistant form of MM mutated in the proteasomal subunit *PSMB5*, responsible for resistance to BTZ and its derivates Ixazomib (IXA) and Carfilzomib (CARF) [4]. CuET potency was indeed unaffected by this mutation, analogous to the p97 inhibitor CB5083 [48]. Importantly, these data place CuET among the promising experimental anti-MM therapeutics such as p97 inhibitors.

The selection pressure imposed by PIs can also trigger adaptive mitigation of the otherwise intense immunoglobulin synthesis, elevated basal ER stress and expanded ER network, characteristic features that underlie the MM cell vulnerability to UPS blockers. Several studies highlighted the importance of immunoglobulin synthesis rate for MM sensitivity to BTZ [21, 24, 34]. Given frequently observed dedifferentiation of MM cells and clonal propagation of pre-plasma cells during therapy [25, 26] the PIs resistance due to decreased immunoglobulin production [19] is clinically relevant. Moreover, this type of MM resistance may explain the observed cross-resistance among different PIs and the limited efficacy of second-generation PIs after the bortezomib-based regimen fails. This current state in the field raises the question of whether any of the UPS inhibitors, in general, may still retain their exceptional potency in such adapted MM cell populations. To test this hypothesis we experimentally mimicked the rescue effect of the decreased proteo-synthesis by concomitant treatment of MM cells with CHX [37], a drug limiting protein translation. This scenario protects MM cells from the toxicity of both BTZ and the p97 inhibitor CB5083. Strikingly, we could not see such CHX-mediated protective effect against treatment by CuET, a rather surprising result given the expected high mechanistic resemblance of CuET to CB5083. Next, we employed the BTZ-adapted AMO1 cell line (AMO1-abzb) as another model [38], in which resistance to BTZ reflects the downregulation of proteo-synthesis (Fig. 4A, 4B). In this BTZ- and partially CB5083- resistant system we confirmed the persistent sensitivity to CuET. Interestingly, in AMO1-abzb cells, both CuET and CB5083 were still capable of inducing the pro-apoptotic factor CHOP, a marker of activated UPR believed to be the main trigger of apoptosis in PI-treated MM cells [34]. Consistent with the role of p97/NPL4 in ER function, these results indicate that both compounds are efficient inducers of UPR, despite the reduced proteo-synthesis and the ensuing reduced proteasome load [37]. Due to endogenous ER stress, MM cells might be more prone to reaching supra-threshold UPR leading to apoptosis, contributing to the exceptional vulnerability to certain drugs. This conclusion is further supported by studies with classical ER-stressors such as tunicamycin and thapsigargin which are capable of UPR induction independently of UPS interference and are very toxic to MM [21, 49].

An important question raised from our data is why CuET is even more efficient in these resistant models than p97 inhibitors. We speculate that this might involve the triggering of additional toxic effects induced by CuET. Upon CuET treatment, NPL4 protein forms aggregates, which leads not only to impairment of protein degradation and p97 function but also activates massive HSR (Fig. 1, ref. [27]). Activated HSR by itself might contribute to the toxic effects in MM cells as suggested in a recent report [50]. Under treatment with CuET, the HSR is activated by NPL4 protein aggregates which can sequester and immobilize various other proteins [31] including the ATR kinase, with consequences for replication stress and DNA damage [32]. Importantly, MM cells display chromosomal abnormalities, a feature possibly linked to the hyperactivated proteolysis with high demands on ubiquitin, the insufficiency of which becomes a limiting factor in the proper function of DNA damage repair [51]. Related to enhanced endogenous stresses, MM also shows overabundance of RNA-DNA hybrids likely reflecting transcription-replication conflicts [52]. Consistent with these previous publications and hypotheses, human myeloma cell lines and a subset of myeloma patients with poor prognosis indeed exhibit high levels of replication stress and DNA damage [53]. High intrinsic DNA damage was confirmed by γH2AX foci across the whole disease spectrum of MGUS to MM [54] including the samples used in our present study (Supplementary Fig. S5). Importantly, MM cells are addicted to the function of ATR making this kinase a new promising therapeutic vulnerability [55]. Thus, CuET might robustly enhance the high endogenous replication stress in MM cells due to the ATR depletion, thereby increasing DNA damage with lethal consequences. Importantly, in this study, we verified the immobilization of not only ATR but also ATM – yet another DNA-damage-related kinase [56] in insoluble fractions from CuET-treated AMO1 and MM1.S cells (Supplementary Fig. S6). These data suggest that besides affecting the cellular proteolytic machinery upstream of the proteasome, direct ER stress induction, and UPR activation, CuET evokes multiple mechanisms that target additional vulnerabilities of MM cells.

From the translational perspective, our data suggest that CuET works as an efficient anti-MM drug independently of the disease stage and resistance status. This notion is supported by our analyses of patients’ bone marrow aspirates using an ex-vivo cytotoxicity test allowing direct comparison of the toxic effect of CuET on MM (CD319^+^/CD138^+^ positive) *vs* normal plasma cells. This approach mimics the physiological environment since besides red blood cells the samples contain all blood and stromal cells and signaling molecules that might affect the therapy [57]. We examined 14 MM patient samples including newly diagnosed, under therapy, and resistant/relapsed. The number of viable CD319^+^/CD138^+^ MM cells was greatly reduced after treatment with CuET in a concentration-dependent manner, regardless of the patient donor or his/her stage of the disease. Importantly, CuET induced its cytotoxic effect preferentially in tumor cells (CD319^+^/CD138^+^). CuET efficiency was also confirmed in primary MM cells from patients resistant and/or relapsed after previous therapies with clinically used classes of drugs.

Altogether, our data suggest that DSF, particularly when combined with copper, is a candidate for an attractive and readily available treatment option for patients with relapsed and/or therapy-resistant MM. Gaps in our knowledge about the pharmacokinetics of CuET formation in patients, interference with other medications and the lack of predictive biomarkers currently preclude a wider application of DSF in oncology. On the other hand, some of the outstanding questions will hopefully be answered by the currently ongoing clinical trials with DSF repurposing in treatment of solid human malignancies, thereby also paving the way for use in therapy-refractory MM patients.

## Materials and methods

### Cell lines

Human multiple myeloma cell lines AMO1 (ATCC) and MM1.S (ATCC) were cultured in RPMI-1640 medium (Thermo Fisher Scientific) with 10% fetal bovine serum (Thermo Fisher Scientific) and 1% penicillin/streptomycin (Sigma-Aldrich). The wild type (WT) and BTZ-resistant AMO1 and L363 myeloma cell lines with point mutations in PSMB5 (A20T, M45I) as well as BTZ- adapted AMO1 abzb cell lines were provided by Departement of Hematology, Kantonsspital St Gallen, St Gallen, Switzerland [4, 38]. The human osteosarcoma cell lines U2OS (ATCC) and breast cancer cell line MDA-MB-231 (ATCC) were cultured in DMEM (Dulbecco’s Modified Eagle Medium, Lonza) with the same supplements as above. All cell lines were cultured under 5% CO2 at 37 °C and tested for mycoplasma contamination.

### XTT Viability assay of multiple myeloma cell lines

Approx. 10,000 cells were seeded per well in a 96-well plate. The next day, cells were treated as described in the figures. The XTT assay was performed according to the manufacturer’s instructions (Applichem). The dye intensity was measured at the 475 nm wavelength using a spectrometer (TECAN, Infinite M200PRO).

### Western blot

The whole-cell lysates were prepared by direct cell lysis in 1× Laemmli sample buffer (1× LSB). The insoluble pellet fraction used for the analysis of immobilized K48-Ubiquitin (Fig. 4D) was obtained by a quick wash of cells with 0.5% Triton X-100 followed by direct cell lysis in 1× LSB. Cell lysates were separated by SDS-PAGE on either hand-cast gels or pre-casted 4–12% gradient gels (SIGMA) and then transferred onto a nitrocellulose membrane. Blocking of the membrane, incubation with primary antibodies, detection with and visualization of secondary antibodies was performed as described previously [31]. Uncropped western blot results are included in the supplementary material.

### Cell fractionation for Triton X insoluble pellets

Myeloma suspension cells were treated as indicated and the Triton X-100 soluble and insoluble MM cell fractions were collected by a procedure as described previously [31].

### Immunofluorescence

Cells grown on coverslips were treated with compounds at indicated concentrations and subsequently either pre-extracted (0.1% Triton X 100 in PBS, for 2 min) and/or fixed with 4% paraformaldehyde for 15 min at room temperature, washed with PBS, and permeabilized with 0.5% Triton X-100 in PBS for 5 min. The following steps, including primary, secondary antibody, and DNA staining were performed as described previously [31]. Samples were visualized using LSM 980 Zeiss Axioimager Z.1 microscope.

### OPP Protein synthesis assay

Firstly, O-propargyl-puromycin (OPP; 20 μM) (Life Technologies) was added to the cells and incubated for 30 min. Next, cells were washed in ice-cold PBS, fixed, permeabilized by 0.5% Triton X 100 in PBS, and stained as described in the manufacturer´s instructions (Click-iT™ Plus OPP Alexa Fluor™ 488 Protein Synthesis Assay Kit). Suspension AMO1 cells were fixed and cytospinned using the Cyto-Tek Sakura instrument (Sakura Finetek, Torrance, CA, USA) onto microscopic slides, followed by permeabilization and staining as described in the manufacturer´s instructions (Click-iT™ Plus OPP Alexa Fluor™ 488 Protein Synthesis Assay Kit). The fluorescent signal depicting active proteosynthesis was acquired via U-plan S Apochromat 40× /0.9 NA objective using an automated microscopic acquisition system (ScanR, Olympus). The signal data from individual cells were processed in the STATISTICA 13.

### Processing of MM patients’ bone marrow aspirates for cell viability assays

Bone marrow (BM) aspirates from multiple myeloma patients were obtained by routine prognostic procedure at the Department of Hemato-oncology of the Faculty Hospital Olomouc (Czech Republic). Informed consent in accordance with the Declaration of Helsinki was obtained from all patients, and approval was obtained from the Ethics Committee of the University Hospital and the Faculty of Medicine Palacky University in Olomouc. Immediately after bone marrow aspiration, samples were mixed with 5 ml of RPMI-1640 containing 100U/ml heparin to prevent coagulation. The percentage of MM cells was identified by flow cytometry using anti-CD-319-PE and anti-CD-139-APC antibodies. The rest of the patients’ bone marrow samples were passed through a 100 μM filter and spin down for 10 min at 20 °C and 445 G. For red blood cells removal, the cell pellets were re-suspended in pre-chilled red blood cell lysis buffer (RBC) (0.15MNH4Cl; 10 mM KHCO3; 0.1 mM EDTA; pH 7.3). The white blood cells including multiple myeloma plasma cells were re-suspended in RPMI-1640 and used for the cell viability experiments (see below).

### Viability assay of MM patients’ samples and flow cytometry analysis

Cells were seeded in duplicates in concentration 1×10^5^ cells/100 μl per well on 96 well plates and treated by CuET (100 and 500 nM), and Mock (0.5% DMSO). After 24 h, CuET- and Mock-treated cells were incubated with CD319-PE antibody for 30 min. After adding 1 ml of RPMI-1640 per sample, cells were centrifuged for 5 min at 200 G. The cell pellets were then re-suspended in RPMI-1640 with DAPI in concentration 0,5 μg/ml and incubated in dark for 15 min. Stained samples were analyzed by flow cytometry using BD FACS Verse (BD Biosciences) and at least 10 000 events were acquired per sample. Collected data were processed using BD FACS Suite (BD Biosciences).

### Processing of MM patient´s bone marrow aspirates for Western blot analysis

First steps of MM patients’ BM aspirates processing were identical to those described above in “Processing of MM patients’ bone marrow aspirates for cell viability assays”. After red blood cells deprivation, samples were filtered through a 100 μM filter and centrifuged at 445 G for 10 min at 20 °C. Cell pellets were re-suspended in separation buffer (auto MACS Separation-Running Buffer, MACS Miltenyi Biotec). To obtain a single-cell suspension before magnetic labeling, cells were passed through 30 μM nylon mesh. Individual cells were magnetically labeled using CD138 microbeads (MACS Miltenyi Biotec) according to the manufacturer´s instructions. Afterward, CD138 positive and CD138 negative cells were lysed in 1× LSB.

### Patient´s samples characteristics

BM samples were derived from 14 patients (6 men and 8 women) in the age of 52-78 years either diagnosed for MM driving stage- monoclonal gammopathy of undetermined significance (MGUS) or Multiple Myeloma (MM). Bone marrow (BM) aspirates from MM patients were obtained by routine prognostic procedure at the Department of Hemato-oncology of the Faculty Hospital Olomouc (Czech Republic) after an appropriate informed consent was signed. Six of the analyzed patients’ samples were freshly diagnosed for MM having no passed therapy, 1 patient was under Velcade (BTZ)-containing therapy, 5 patients were clinically diagnosed as relapsed after combinatory Velcade (BTZ), Revlimid (lenalidomide)/Thalidomid (thalidomide), Dexamethasone or Velcade (BTZ), Cytoxan (Cyclophosphamide), Dexamethasone therapy. One patient was resistant to BTZ as well as to combinatory therapy by Velcade (BTZ), Revlimid (lenalidomide), and Dexamethasone.

### Antibodies and chemicals

The following antibodies were used for immunoblotting: anti-ubiquitin lys48-specific (Merck Millipore, clone Apu2), anti-VCP (Novus Bio, NBP100-1557), anti-NPLOC4 (Novus Bio, NBP1-82166), HSP70 (Enzo, ADI-SPA-830), anti-XBP1 (Santa Cruz Biotechnology, sc-7160), ATF4 (Merck Millipore, ABE387), 1D4), anti-lamin B (Santa Cruz Biotechnology, sc-6217), MEK-1 Antibody (C-18) (Santa Cruz Biotechnology, sc-219), anti SMC1 antibody (Abcam, ab1276-50), CHOP (L63F7) (Cell Signaling, #2895), Caspase-3 antibody (Cell Signaling, #9662 S), Cleaved PARP (Asp214) antibody (Cell Signaling, #9544), Anti-GRP78 BiP antibody (Abcam,ab21685), ATR antibody (N-19) (Santa Cruz Biotechnology, sc-1887), ATM (D2E2) (Cell Signaling, #2873), Phospho-Histone H2A.X (Ser139) (20E3) (Cell Signaling, #9718), IRF-4 antibody (Cell Signaling, #4964). For immunofluorescence was the anti-ubiquitin lys48-specific antibody (Merck Millipore, clone Apu2), HSP70 antibody (Enzo, ADI-SPA-830). The antibodies used for detection of myeloma cells by flow cytometry measurement include PE anti-human CD319 (CRACC) antibody (BioLegend, 331806) and APC anti-human CD138 (44F9) antibody (Miltenyi Biotec, 130-117-544).

Chemicals used in this study: CuET (bis-diethyldithiocarbamate-copper complex, TCI chemicals), disulfiram (Sigma, St. Louis, MO, USA), bortezomib (PS-341, Sigma-Aldrich), CB5083 (Selleckchem), cycloheximide (Sigma-Aldrich).

## Supplementary information

Supplemental Figures

Uncropped western blots

Checklist

## Data Availability

The data used to support the findings of this study are available from the corresponding author upon request.

## References

[1] Anderson KC (2004). Bortezomib therapy for myeloma. Curr Hematol Rep..

[2] Greenlee RT, Murray T, Bolden S, Wingo PA (2000). Cancer statistics, 2000. CA Cancer J Clin..

[3] Allmeroth K, Horn M, Kroef V, Miethe S, Müller R-U, Denzel MS (2021). Bortezomib resistance mutations in PSMB5 determine response to second-generation proteasome inhibitors in multiple myeloma. Leukemia.

[4] Barrio S, Stühmer T, Da-Viá M, Barrio-Garcia C, Lehners N, Besse A (2019). Spectrum and functional validation of PSMB5 mutations in multiple myeloma. Leukemia.

[5] Li J, Yakushi T, Parlati F, Mackinnon AL, Perez C, Ma Y (2017). Capzimin is a potent and specific inhibitor of proteasome isopeptidase Rpn11. Nat Chem Biol.

[6] D’Arcy P, Brnjic S, Olofsson MH, Fryknäs M, Lindsten K, De Cesare M (2011). Inhibition of proteasome deubiquitinating activity as a new cancer therapy. Nat Med..

[7] Anchoori RK, Karanam B, Peng S, Wang JW, Jiang R, Tanno T (2013). A bis-benzylidine piperidone targeting proteasome ubiquitin receptor RPN13/ADRM1 as a therapy for cancer. Cancer Cell..

[8] Song Y, Ray A, Li S, Das DS, Tai YT, Carrasco RD (2016). Targeting proteasome ubiquitin receptor Rpn13 in multiple myeloma. Leukemia.

[9] Song Y, Li S, Ray A, Das DS, Qi J, Samur MK (2017). Blockade of deubiquitylating enzyme Rpn11 triggers apoptosis in multiple myeloma cells and overcomes bortezomib resistance. Oncogene.

[10] Tian Z, D’Arcy P, Wang X, Ray A, Tai Y-T, Hu Y (2014). A novel small molecule inhibitor of deubiquitylating enzyme USP14 and UCHL5 induces apoptosis in multiple myeloma and overcomes bortezomib resistance. Blood.

[11] Hyer ML, Milhollen MA, Ciavarri J, Fleming P, Traore T, Sappal D (2018). A small-molecule inhibitor of the ubiquitin activating enzyme for cancer treatment. Nat Med.

[12] Anderson DJ, Le Moigne R, Djakovic S, Kumar B, Rice J, Wong S (2015). Targeting the AAA ATPase p97 as an approach to treat cancer through disruption of protein homeostasis. Cancer Cell.

[13] Zhuang J, Shirazi F, Singh RK, Kuiatse I, Wang H, Lee HC (2019). Ubiquitin-activating enzyme inhibition induces an unfolded protein response and overcomes drug resistance in myeloma. Blood.

[14] Le Moigne R, Aftab BT, Djakovic S, Dhimolea E, Valle E, Murnane M (2017). The p97 Inhibitor CB-5083 Is a Unique Disrupter of Protein Homeostasis in Models of Multiple Myeloma. Mol Cancer Ther.

[15] Lichter DI, Danaee H, Pickard MD, Tayber O, Sintchak M, Shi H (2012). Sequence analysis of β-subunit genes of the 20S proteasome in patients with relapsed multiple myeloma treated with bortezomib or dexamethasone. Blood.

[16] Politou M, Karadimitris A, Terpos E, Kotsianidis I, Apperley JF, Rahemtulla A (2006). No evidence of mutations of the PSMB5 (beta-5 subunit of proteasome) in a case of myeloma with clinical resistance to Bortezomib. Leuk Res.

[17] Corre J, Cleynen A, Robiou du Pont S, Buisson L, Bolli N, Attal M (2018). Multiple myeloma clonal evolution in homogeneously treated patients. Leukemia.

[18] Ziccheddu B, Biancon G, Bagnoli F, De Philippis C, Maura F, Rustad EH (2020). Integrative analysis of the genomic and transcriptomic landscape of double-refractory multiple myeloma. Blood Adv.

[19] Leung-Hagesteijn C, Erdmann N, Cheung G, Keats JJ, Stewart AK, Reece DE (2013). Xbp1s-negative tumor B cells and pre-plasmablasts mediate therapeutic proteasome inhibitor resistance in multiple myeloma. Cancer Cell.

[20] Cenci S, Mezghrani A, Cascio P, Bianchi G, Cerruti F, Fra A (2006). Progressively impaired proteasomal capacity during terminal plasma cell differentiation. EMBO J.

[21] Ling SCW, Lau EKK, Al-Shabeeb A, Nikolic A, Catalano A, Iland H, et al. Response of myeloma to the proteasome inhibitor bortezomib is correlated with the unfolded protein response regulator XBP-1. 1. 2012;97:64–72. 10.3324/haematol.2011.043331 PMC3248932 21993678

[22] Zang M, Guo J, Liu L, Jin F, Feng X, An G (2020). Cdc37 suppression induces plasma cell immaturation and bortezomib resistance in multiple myeloma via Xbp1s. Oncogenesis.

[23] Qin X-Q, An G, Li Z-J, Liu L-T, Xu Y, Yang L-H (2019). Secretory status of monoclonal immunoglobulin is related to the outcome of patients with myeloma: a retrospective study. Blood Adv.

[24] Meister S, Schubert U, Neubert K, Herrmann K, Burger R, Gramatzki M (2007). Extensive immunoglobulin production sensitizes myeloma cells for proteasome inhibition. Cancer Res.

[25] Paiva B, Puig N, Cedena MT, de Jong BG, Ruiz Y, Rapado I (2017). Differentiation stage of myeloma plasma cells: biological and clinical significance. Leukemia.

[26] Chaidos A, Barnes CP, Cowan G, May PC, Melo V, Hatjiharissi E (2013). Clinical drug resistance linked to interconvertible phenotypic and functional states of tumor-propagating cells in multiple myeloma. Blood.

[27] Xu Y, Zhou Q, Feng X, Dai Y, Jiang Y, Jiang W (2020). Disulfiram/copper markedly induced myeloma cell apoptosis through activation of JNK and intrinsic and extrinsic apoptosis pathways. Biomed Pharmacother.

[28] Meier S, Cantilena S, Niklison Chirou MV, Anderson J, Hargrave D, Salomoni P (2021). Alcohol-abuse drug disulfiram targets pediatric glioma via MLL degradation. Cell Death Dis.

[29] Chen D, Cui QC, Yang H, Dou QP (2006). Disulfiram, a clinically used anti-alcoholism drug and copper-binding agent, induces apoptotic cell death in breast cancer cultures and xenografts via inhibition of the proteasome activity. Cancer Res.

[30] Yoshino H, Yamada Y, Enokida H, Osako Y, Tsuruda M, Kuroshima K (2020). Targeting NPL4 via drug repositioning using disulfiram for the treatment of clear cell renal cell carcinoma. PLoS ONE.

[31] Skrott Z, Mistrik M, Andersen KK, Friis S, Majera D, Gursky J (2017). Alcohol-abuse drug disulfiram targets cancer via p97 segregase adaptor NPL4. Nature.

[32] Majera D, Skrott Z, Chroma K, Merchut-Maya JM, Mistrik M, Bartek J. Targeting the NPL4 Adaptor of p97/VCP Segregase by Disulfiram as an Emerging Cancer Vulnerability Evokes Replication Stress and DNA Damage while Silencing the ATR Pathway. Cells. 2020;18:9. 10.3390/cells9020469 PMC7072750 32085572

[33] Dai RM, Li CC (2001). Valosin-containing protein is a multi-ubiquitin chain-targeting factor required in ubiquitin-proteasome degradation. Nat Cell Biol.

[34] Obeng EA, Carlson LM, Gutman DM, Harrington WJ, Lee KP, Boise LH (2006). Proteasome inhibitors induce a terminal unfolded protein response in multiple myeloma cells. Blood.

[35] Shah JJ, Orlowski RZ (2009). Proteasome inhibitors in the treatment of multiple myeloma. Leukemia.

[36] Skrott Z, Majera D, Gursky J, Buchtova T, Hajduch M, Mistrik M (2019). Disulfiram’s anti-cancer activity reflects targeting NPL4, not inhibition of aldehyde dehydrogenase. Oncogene.

[37] Cenci S, Oliva L, Cerruti F, Milan E, Bianchi G, Raule M (2012). Pivotal Advance: protein synthesis modulates responsiveness of differentiating and malignant plasma cells to proteasome inhibitors. J Leukoc Biol.

[38] Rückrich T, Kraus M, Gogel J, Beck A, Ovaa H, Verdoes M (2009). Characterization of the ubiquitin–proteasome system in bortezomib-adapted cells. Leukemia.

[39] O’Connell FP, Pinkus JL, Pinkus GS (2004). CD138 (syndecan-1), a plasma cell marker immunohistochemical profile in hematopoietic and nonhematopoietic neoplasms. Am J Clin Pathol.

[40] Frigyesi I, Adolfsson J, Ali M, Christophersen MK, Johnsson E, Turesson I (2014). Robust isolation of malignant plasma cells in multiple myeloma. Blood.

[41] Soh KT, Tario JD, Hahn T, Hillengass J, McCarthy PL, Wallace PK CD319 (SLAMF7) an alternative marker for detecting plasma cells in the presence of daratumumab or elotuzumab. Cytometry Part B, Clinical Cytometry [Internet]. 2020 Oct 6 [cited 2022 Jan 21]; Available from: https://www.meta.org/papers/cd319-slamf7-an-alternative-marker-for-detecting/33017079. 10.1002/cyto.b.21961 PMC8940539 33017079

[42] Zonder JA, Mohrbacher AF, Singhal S, van Rhee F, Bensinger WI, Ding H (2012). A phase 1, multicenter, open-label, dose escalation study of elotuzumab in patients with advanced multiple myeloma. Blood.

[43] Pinto V, Bergantim R, Caires HR, Seca H, Guimarães JE, Vasconcelos MH. Multiple Myeloma: Available Therapies and Causes of Drug Resistance. Cancers (Basel). 2020;12. 10.3390/cancers12020407 PMC7072128 32050631

[44] Meraz-Torres F, Plöger S, Garbe C, Niessner H, Sinnberg T. Disulfiram as a Therapeutic Agent for Metastatic Malignant Melanoma-Old Myth or New Logos? Cancers (Basel). 2020;12. 10.3390/cancers12123538 PMC7760689 33260923

[45] Lipsky JJ, Shen ML, Naylor S (2001). In vivo inhibition of aldehyde dehydrogenase by disulfiram. Chem-Biol Interact.

[46] Pan M, Zheng Q, Yu Y, Ai H, Xie Y, Zeng X (2021). Seesaw conformations of Npl4 in the human p97 complex and the inhibitory mechanism of a disulfiram derivative. Nat Commun.

[47] Conticello C, Martinetti D, Adamo L, Buccheri S, Giuffrida R, Parrinello N (2012). Disulfiram, an old drug with new potential therapeutic uses for human hematological malignancies. Int J Cancer.

[48] Brünnert D, Kraus M, Stühmer T, Kirner S, Heiden R, Goyal P (2019). Novel cell line models to study mechanisms and overcoming strategies of proteasome inhibitor resistance in multiple myeloma. Biochimica et Biophysica Acta (BBA) - Mol Basis Dis.

[49] Borjan B, Kern J, Steiner N, Gunsilius E, Wolf D, Untergasser G (2019). Spliced XBP1 levels determine sensitivity of multiple myeloma cells to proteasome inhibitor bortezomib independent of the unfolded protein response mediator GRP78. Front Oncol.

[50] Sha Z, Goldberg AL (2020). Multiple myeloma cells are exceptionally sensitive to heat shock, which overwhelms their proteostasis network and induces apoptosis. PNAS.

[51] Chroma K, Mistrik M, Moudry P, Gursky J, Liptay M, Strauss R (2017). Tumors overexpressing RNF168 show altered DNA repair and responses to genotoxic treatments, genomic instability and resistance to proteotoxic stress. Oncogene.

[52] Dutrieux L, Lin Y-L, Lutzmann M, Rodriguez R, Cogné M, Pasero P (2021). Transcription/replication conflicts in tumorigenesis and their potential role as novel therapeutic targets in multiple myeloma. Cancers.

[53] Cottini F, Hideshima T, Suzuki R, Tai Y-T, Bianchini G, Richardson PG (2015). Synthetic lethal approaches exploiting DNA damage in aggressive myeloma. Cancer Disco.

[54] Walters DK, Wu X, Tschumper RC, Arendt BK, Huddleston PM, Henderson KJ (2011). Evidence for ongoing DNA damage in multiple myeloma cells as revealed by constitutive phosphorylation of H2AX. Leukemia.

[55] Botrugno OA, Bianchessi S, Zambroni D, Frenquelli M, Belloni D, Bongiovanni L (2020). ATR addiction in multiple myeloma: synthetic lethal approaches exploiting established therapies. Haematologica.

[56] Bartek J, Bartkova J, Lukas J (2007). DNA damage signalling guards against activated oncogenes and tumour progression. Oncogene.

[57] Markovina S, Callander NS, O’Connor SL, Xu G, Shi Y, Leith CP (2010). Bone marrow stromal cells from multiple myeloma patients uniquely induce bortezomib resistant NF-κB activity in myeloma cells. Mol Cancer..

