# Supplementary material for: A drug repurposing strategy for overcoming human multiple myeloma resistance to standard-of-care treatment
Source: Cell Death Dis. 2022 Mar 4;13(3):203. doi: 10.1038/s41419-022-04651-w (PMC8897388; doi:10.1038/s41419-022-04651-w)
Supplement: Supplementary file 1 — Supplemental Figures [file 41419_2022_4651_MOESM1_ESM.docx]

**Supplementary Figures**

**B**

**C**

**A**

**IC50 values after 24 h of CuET treatment**

| **Cell line** | **Type** |  | **IC50 (nM)** | |
| --- | --- | --- | --- | --- |
| AMO-1 | Multiple Myeloma | 113,8 | 115,3 | 118,3 |
| MM1S | Multiple Myeloma | 211,1 | 180,9 | 194,8 |
| MDA-MB-231 | Breast adenocarcinoma | 362,6 | 333,5 | 326,2 |
| U2OS | Osteosarcoma | 295,3 | 341,7 | 311,8 |

**
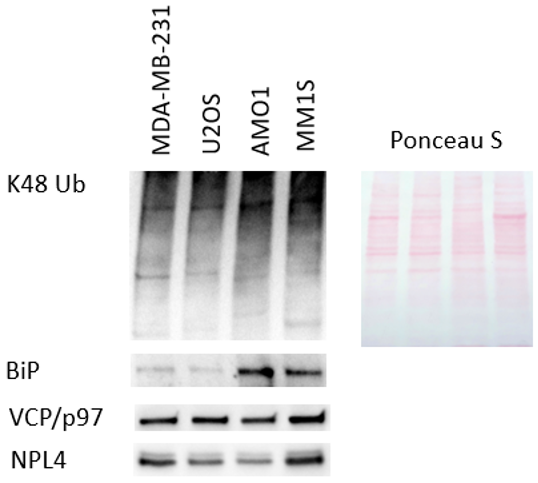
**

**Supplementary Figure 1. Multiple myeloma cell lines are particularly sensitive to Disulfiram´s metabolite CuET. A** CuET cytotoxicity measured by tetrazolium dye ((2,3 bis-(2-methoxy-4nitro-5-sulphenyl)-2h-tetrazolium-5-carboxamide (XTT) test after 24 hours of treatment in human cell lines derived from osteosarcoma (U2OS), breast cancer (MDA-MB-231) and multiple myeloma (AMO1, MM1S). Data are mean+-s.d. of three independent experiments. **B** IC_50_ values from three independent biological experiments documenting different CuET-induced cytotoxicity across the tested panel of cancer cell lines (24 treatment). LD50 values were calculated using Graphpad Prism software based on survival curves from at three independent experiments. **C** Immunoblot detection of the endogenous level of poly-Ub proteins together with CuET target NPL4 and VCP/p97 itself in MDA-MB-231, U2OS, AMO1 and MM1.S cell lines.

**
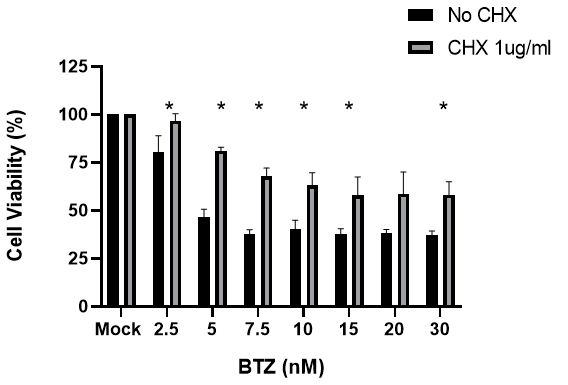
**

**Supplementary Figure 2. Co-treatment with cycloheximide (CHX) does not rescue MM1.S cell death in CuET- treated samples in contrast to BTZ and p97 inhibitor (CB5083).** MM1.S cells were cultured for 24 h in the presence of the indicated doses of CuET, BTZ, and CB5083 in the presence or absence of 1 μg/ml CHX. Data are mean +-s.d. of three independent experiments. Statistical significance was determined using T-test, * P < 0,05.

**
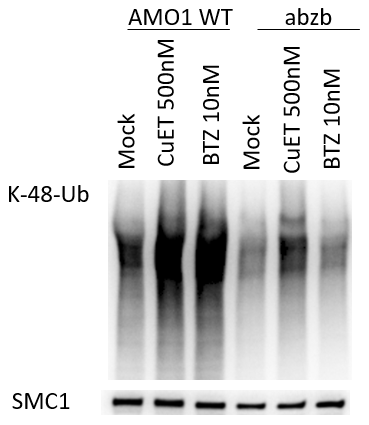
**

**Supplementary Fugure 3. CuET in contrast to BTZ induces accumulation of K48-Ub proteins in AMO1 abzb similarly to AMO1 WT.** BTZ adapted AMO1 abzb and non-adapted AMO1 (WT) cells were treated for 5 hours with indicated concentrations of BTZ and CuET and subsequently lysed and probed for the level of K-48-Ub proteins. SMC1 is used as a loading control.

**
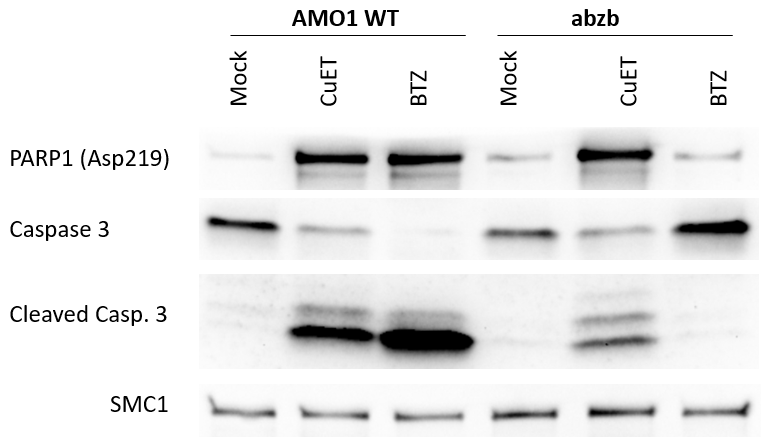
**

**Supplementary Fugure 4. CuET in contrast to BTZ, induces the apoptotic cell death in AMO1 abzb similarly to AMO1 WT.** BTZ adapted AMO1 abzb and non-adapted AMO1 (WT) cells were treated for 24 hours with 250nM CuET and 5nM BTZ and subsequently lysed and probed for the level of cleaved PARP1 (Asp219) and Caspase 3. SMC1 is used as a loading control.

**
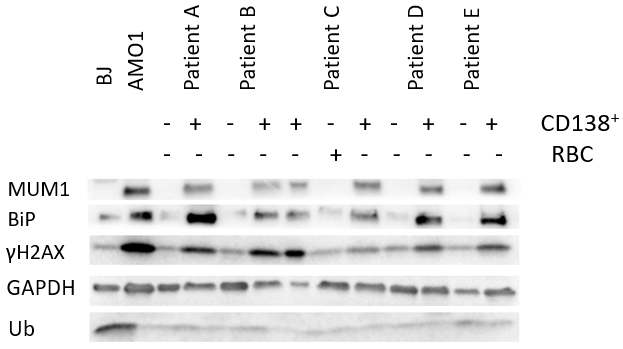
**

**Supplementary Figure 5. Level of endogenous DNA damage measured as γH2AX intensity in cell lysates isolated from primary CD138^+^ MM patients cells compared to rest of B cells.** Multiple myeloma patients´ samples were acquired using the magnetic separation method with CD138^+^ microbeads (Miltenyi Biotec).


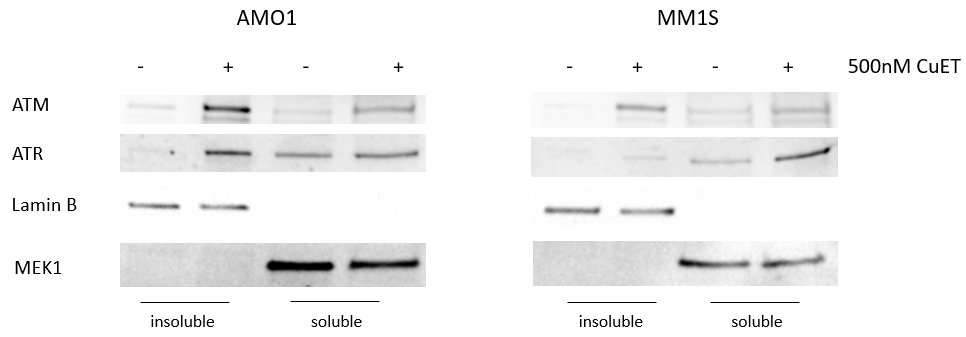


**Supplementary Figure 6. Western blot analysis of CuET- induced immobilization of DDR-associated kinases- ATR and ATM.** Cell fractionated samples of AMO1 and MM1.S cell lines treated 5 hours with 500nM CuET were probed for the level of ATM and ATR. MEK1 and Lamin B were used as loading controls for insoluble and soluble cell fraction.
