# Supplementary material for: A drug repurposing strategy for overcoming human multiple myeloma resistance to standard-of-care treatment
Source: Cell Death Dis. 2022 Mar 4;13(3):203. doi: 10.1038/s41419-022-04651-w (PMC8897388; doi:10.1038/s41419-022-04651-w)

Original data of western blot for Figure 1 A

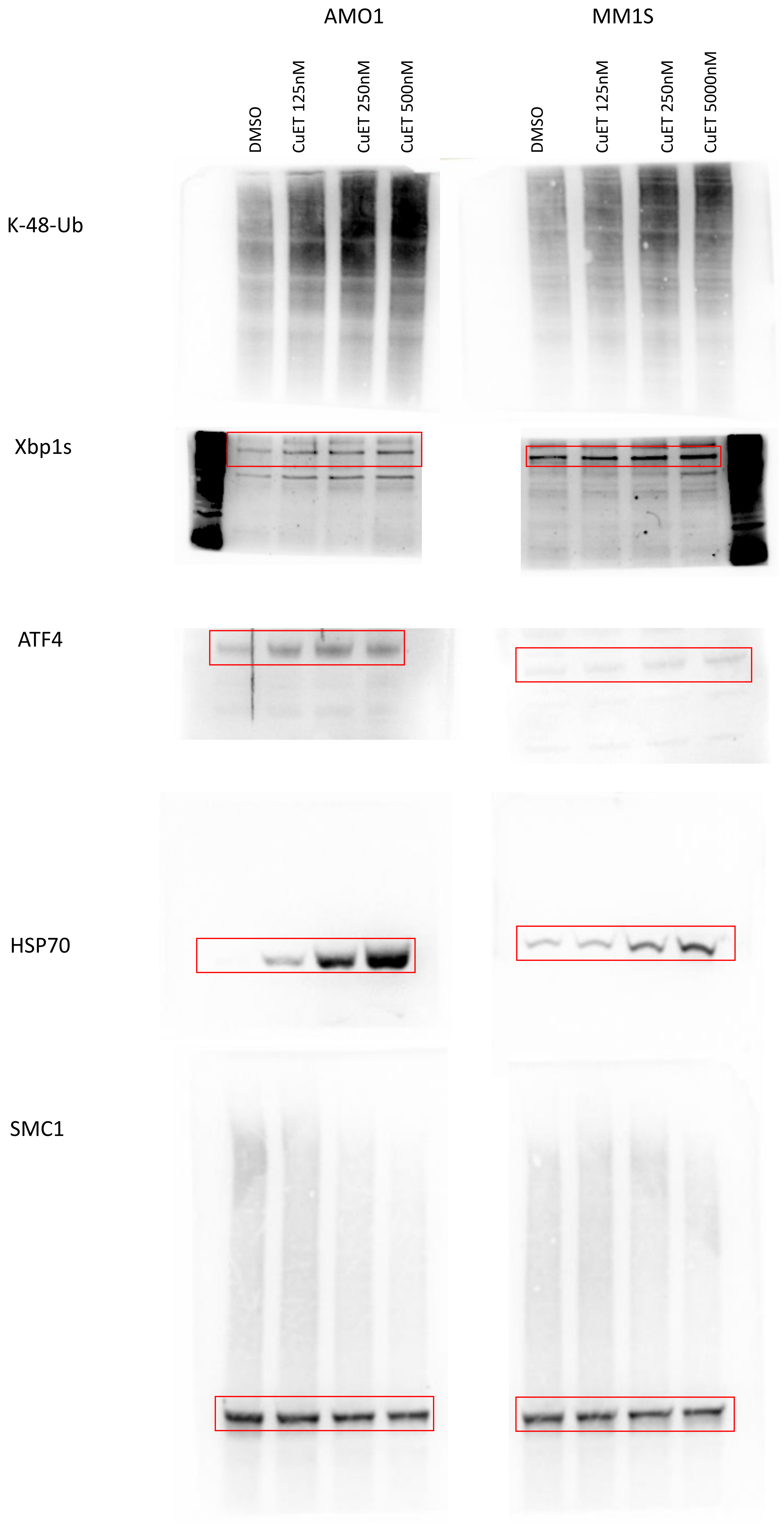

Original data of western blot for Figure 1 B

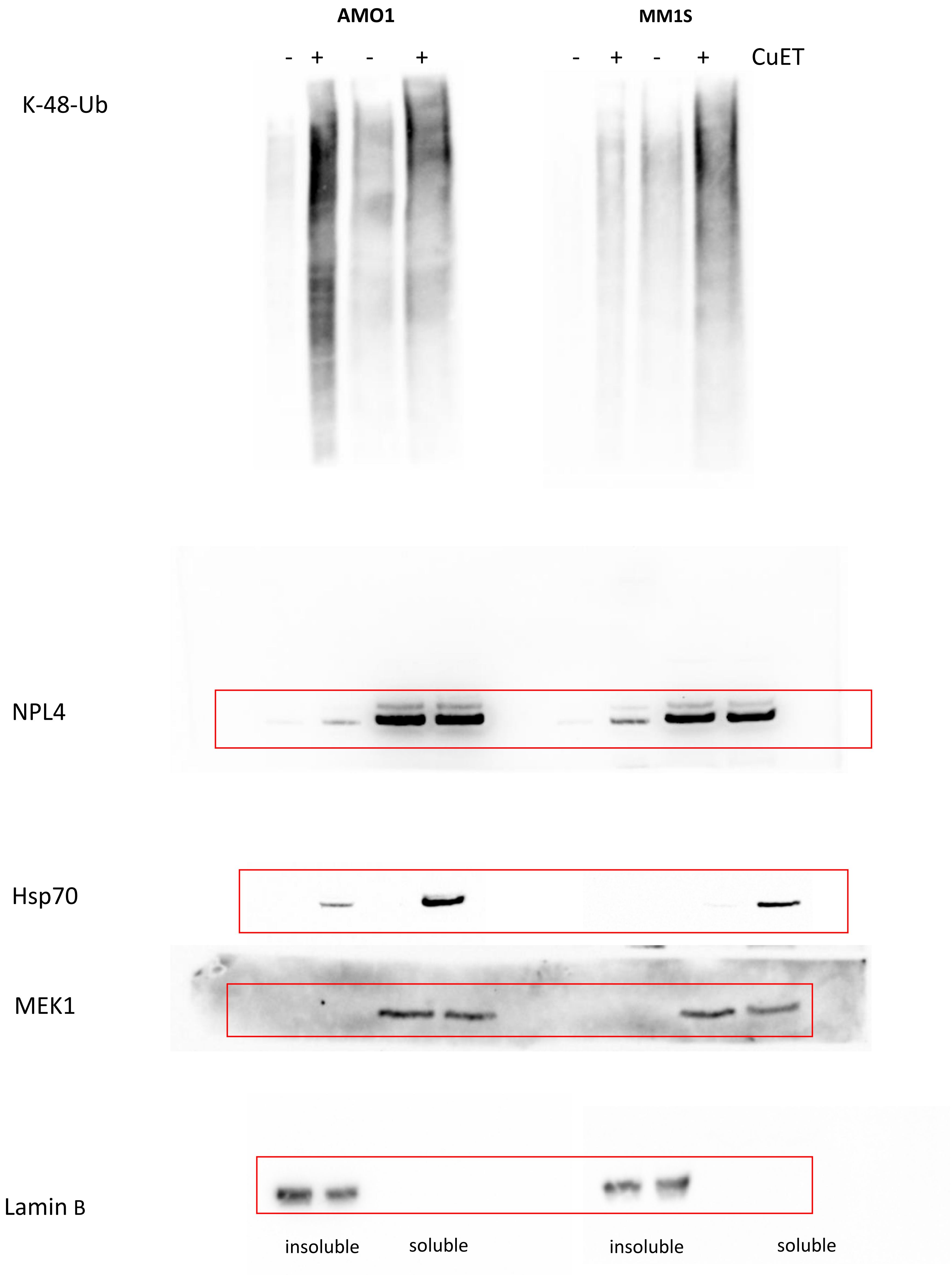

Original data of western blot for Figure 2 B

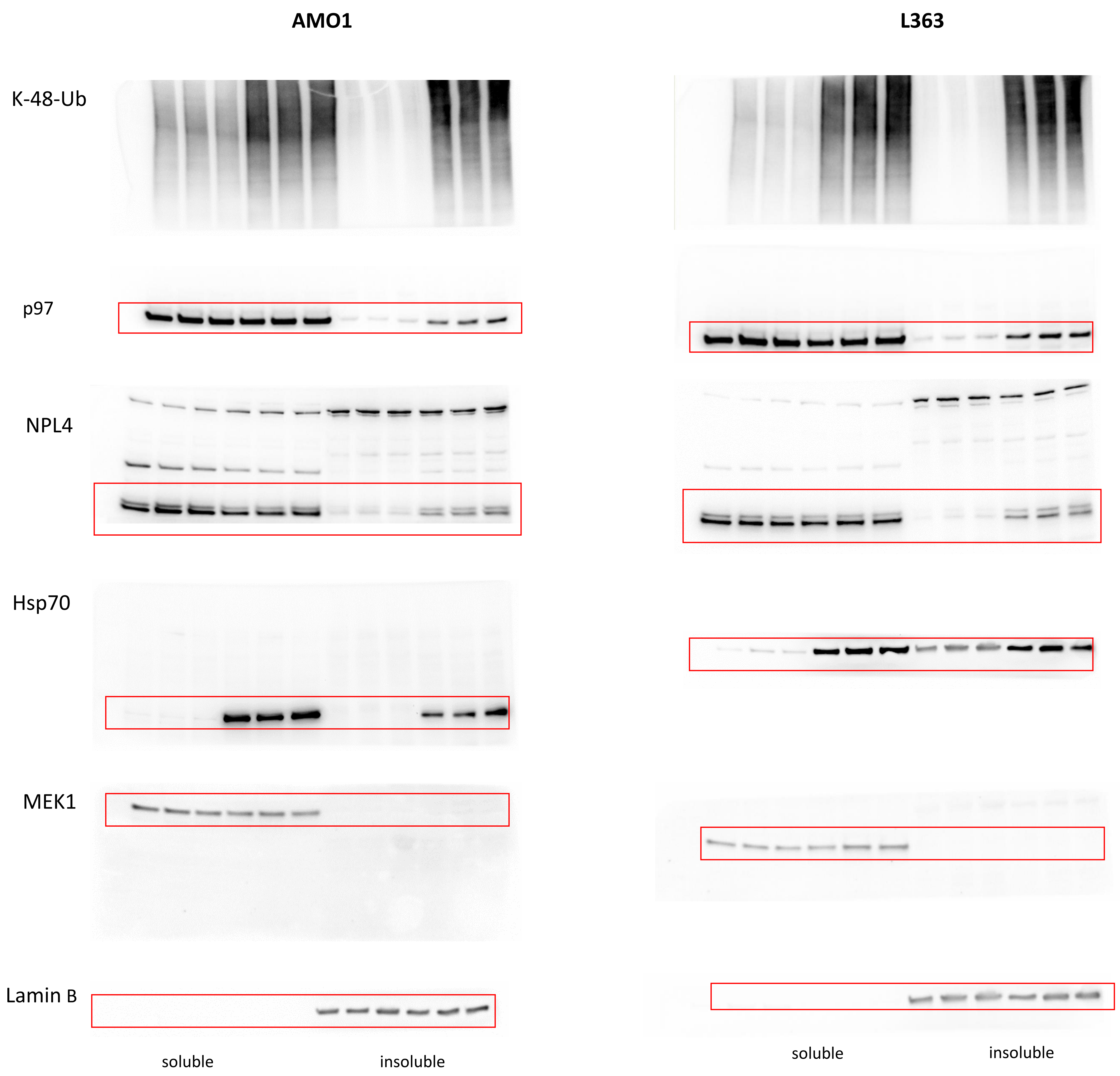

Original data of western blot for Figure 4

C

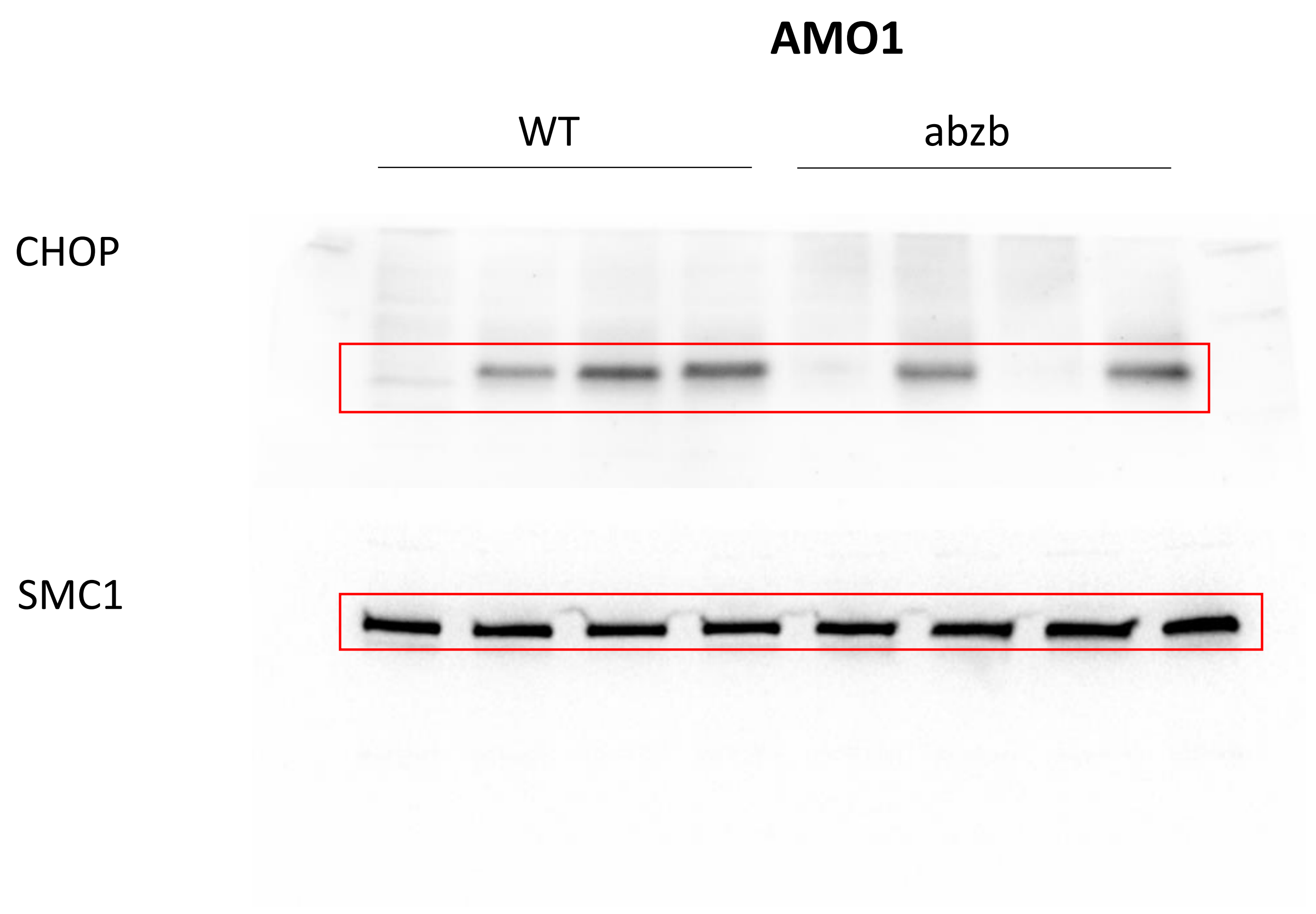

D

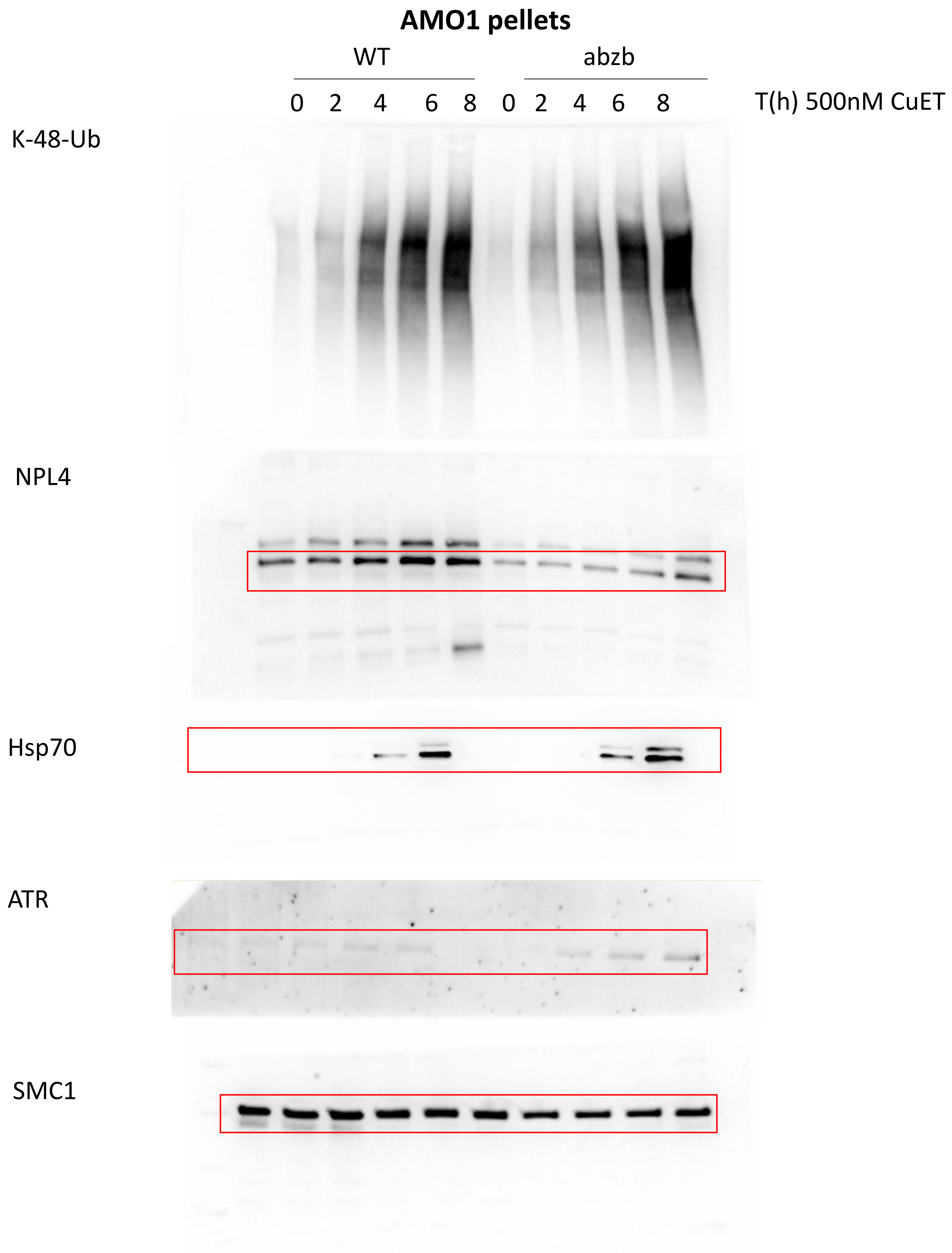

Original data of western blot for Supplementary Figure 1 C

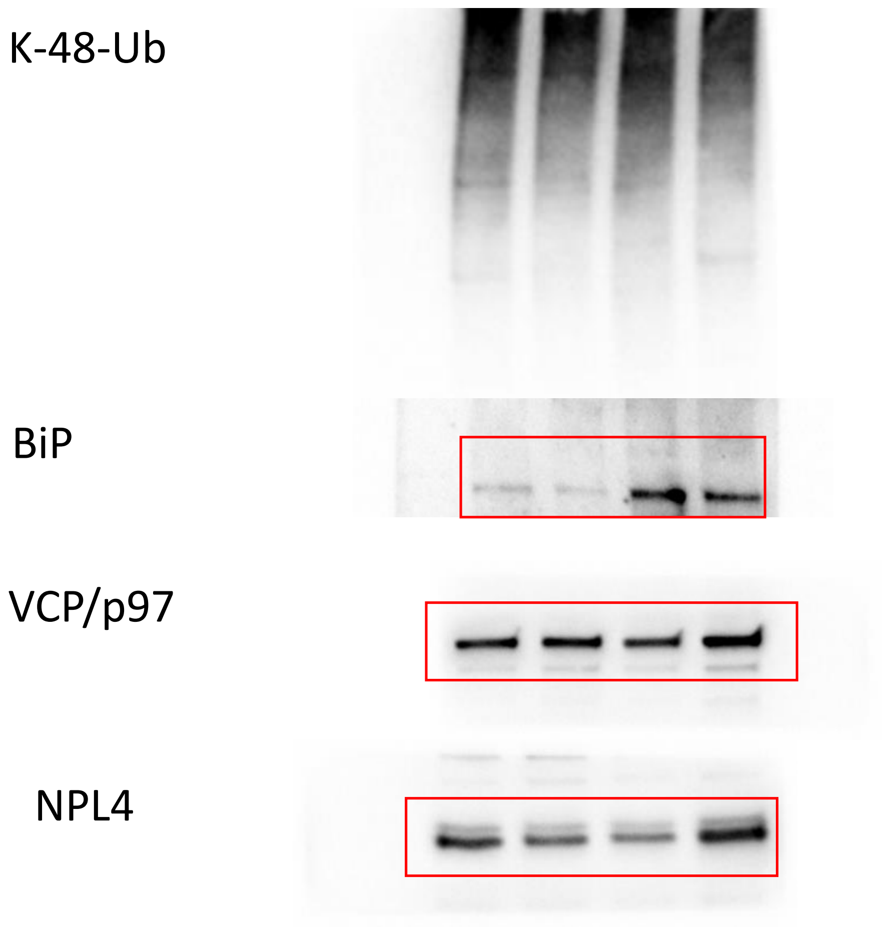

Original data of western blot for Supplementary Figure 3

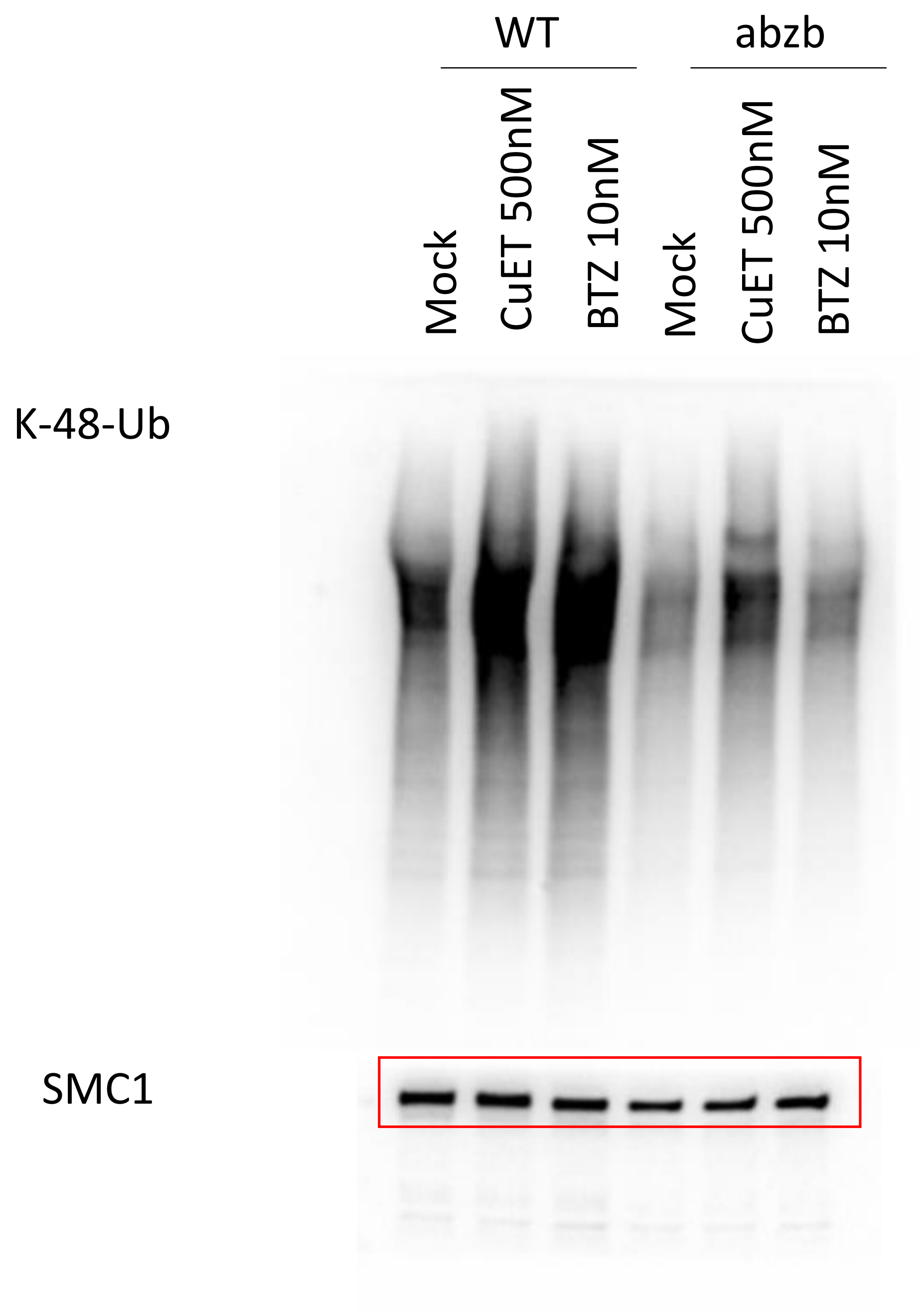

Original data of western blot for Supplementary Figure 4

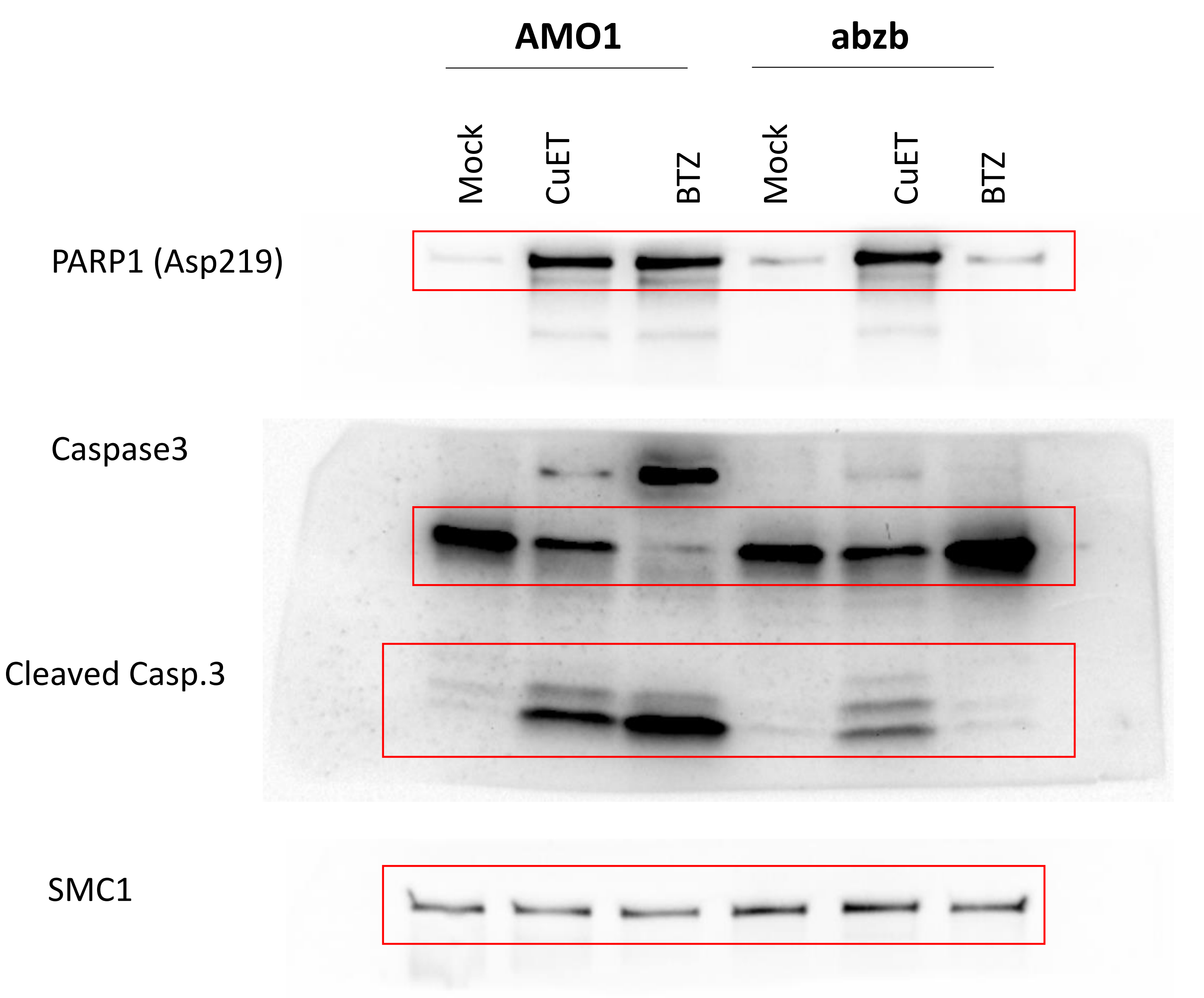

Original data of western blot for Supplementary Figure 5

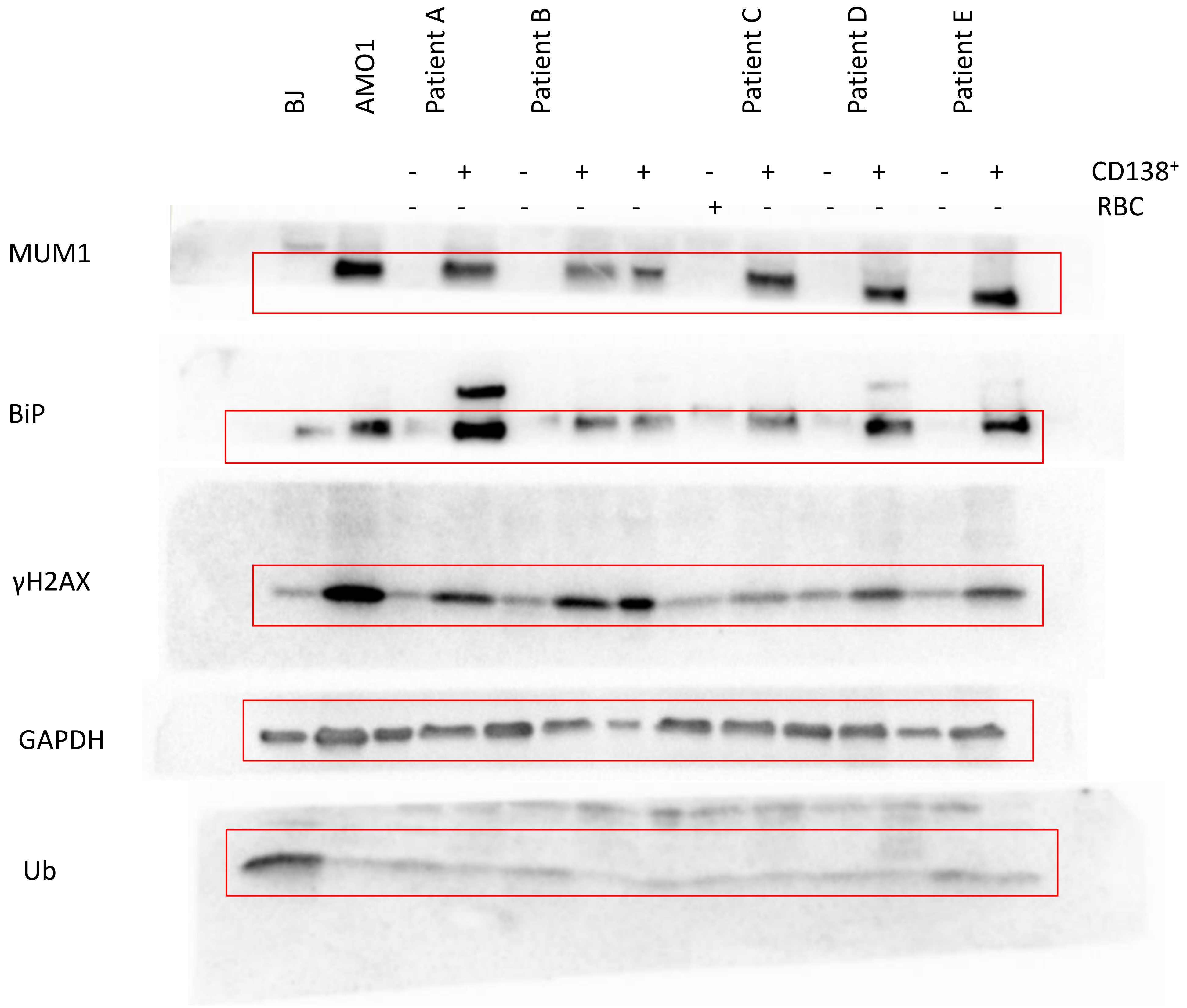

Original data of western blot for Supplementary Figure 6

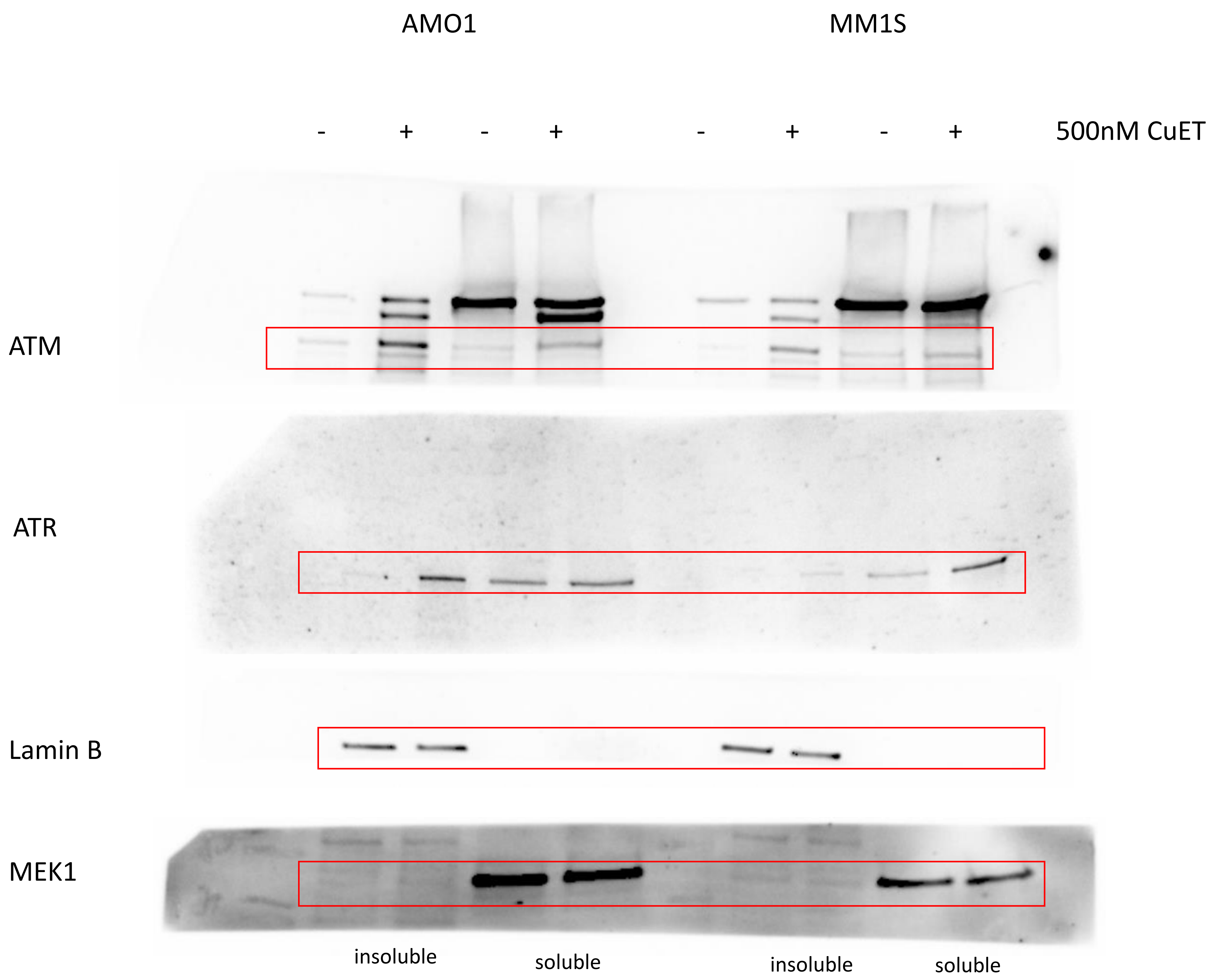

Supplement: Supplementary file 2 — Uncropped western blots [file 41419_2022_4651_MOESM2_ESM.pdf]
